# Geometry-Driven Deformation and Degradation Behavior of Crimped Electrical Connections Under Coupled Environmental and Chemical Loading

**DOI:** 10.3390/ma19112342

**Published:** 2026-06-01

**Authors:** Cevher Sunguray, Satılmış Ürgün, Sinan Fidan, Mustafa Özgür Bora

**Affiliations:** Department of Aerospace Engineering, Faculty of Engineering, Kocaeli University, 41001 Kocaeli, Türkiye; urgun@kocaeli.edu.tr (S.Ü.); sfidan@kocaeli.edu.tr (S.F.); ozgur.bora@kocaeli.edu.tr (M.Ö.B.)

**Keywords:** crimp geometry, contact mechanics, electrical contact behavior, environmental degradation, scanning electron microscopy (SEM), finite element modeling (FEM)

## Abstract

Crimped electrical connections must maintain electrical continuity and mechanical load transfer capability under combined environmental and operational stressors throughout their service life. Although the environmental durability of electrical connectors has been extensively studied, previous studies have mainly focused on material, environmental, or electrical effects in isolation, whereas the coupled influence of crimp geometry on electrical–mechanical degradation and contact evolution remains insufficiently understood. In this study, crimp geometry was isolated as the primary independent variable to investigate geometry-driven degradation behavior in crimped connections. Three crimp configurations (Type A, Type B, and Type C) were subjected to temperature cycling (−55 °C to +70 °C), high humidity (90–95% RH), and combined chemical–electrical loading conditions involving representative fluids and short-circuit current. Electrical and mechanical responses were evaluated using relative resistance variation ΔR (%) and tensile strength change ΔT (%), while factorial ANOVA quantified parameter contributions. The results indicate that crimp geometry dominates the response under thermal–humidity exposure, whereas the chemical exposure type becomes the governing factor for electrical degradation under coupled chemical–electrical conditions. SEM analysis reveals that geometry-dependent plastic deformation governs contact continuity and void formation, leading to a transition from continuous conductive networks to fragmented contact structures. These findings are further supported by FEM analyses, which provide qualitative insight into the deformation response as a function of the geometric parameters. This work presents a geometry-based experimental framework for understanding the degradation behavior of crimped bonding structures under dual-exposure test conditions.

## 1. Introduction

The geometric parameters involved in aircraft bonding and grounding joints are critical not only for maintaining electrical and mechanical connectivity over their lifetime but also in facilitating the dissipation of lightning currents and ensuring electromagnetic compatibility. Although organizations such as SAE International, FAA, and EASA provide specifications for the allowable level of resistance for bonding joints, most studies have focused on operational requirements rather than on the physical mechanisms responsible for degradation under combined loading conditions [1,2,3,4].

The geometric configuration of the crimp interface is recognized as one of the primary factors governing the contact surface area, the properties of plastic deformation, and the force transmission capabilities, which are among the criteria determining the reliability of an electric connector. Prior research has demonstrated that differences in the level of crimping depth and energy of deformation influence the tensile strength and contact resistance. For example, Li et al. showed that tensile strength improvement was directly associated with crimp deformation [5]. Furthermore, Li and Fan further established that the failure strength of terminals depended on the energy of deformation at the conductor–terminal interface [6]. Also, Nguyen et al. found that an increase in the contact surface area with crimping parameters significantly reduced the contact resistance [7].

The geometry of the crimped joint itself controls the level of conductor containment, local contact pressure distribution, and plastic deformation of the conductor bundle. The interplay between these properties defines the amount of real surface contact and continuity of the conductive path and hence determines the level of efficiency for current transfer and mechanical load transfer as well. In the face of environmental stresses, such as heating and cooling, moisture presence, vibrations, and chemical attack, geometries that are not properly compacted or are unevenly deformed tend to form voids, stress concentrations, and poor contact interfaces, progressively increasing electrical resistance and reducing mechanical integrity. The effects of both environmental stress and crimp interface geometry on the degradation process are not yet well understood.

Environmental parameters such as cycling temperature and high humidity are well known to accelerate metal surface degradation. High humidity levels increase ionic transport, thus making electrical contact resistance unstable [7,8,9]. The impacts become more pronounced when there is discontinuity in the metal coating surfaces and metal surface porosity. Importantly, the extent of environmental degradation depends strongly on interfacial characteristics, which depend on the geometric and metallurgical properties of the metal crimping surface. However, despite the environmental degradation factors, current studies that analyze the impact of the environment on electrical contacts do not address any potential changes in crimp geometry.

Chemical exposure is yet another category of operational stress seen in aircraft. Contact with fluids such as Jet A-1, Skydrol, and Type I de-icing fluids results in oxidation, galvanic reactions, and pitting corrosion at contact interfaces. Elevated contact resistance under chemically assisted corrosion has been reported in previous studies [10,11,12,13]. However, there is an area where little is known about the effect of crimp geometry on both environmental and chemical exposure.

According to recent research, connector reliability cannot be attributed merely to assembly quality or to parameter-based analysis alone. Furthermore, exposure-induced surface degradation processes have been found to exhibit highly nonlinear degradation behaviors that strongly depend on local deformation patterns and the integrity of contact conditions [14]. This implies that degradation in crimped electrical connections is governed by exposure–geometry interactions rather than by individual parameter effects.

However, a comprehensive study that considers both crimp structure, exposure to the environment, exposure to chemicals, and the impact of such coupled phenomena on electrical and mechanical properties remains absent from the literature.

In the context of crimped electrical connections, crimp force, crimp depth, and crimp quality are frequently treated in the literature as process-control or inspection parameters associated with assembly conformity. In contrast, the present study focuses specifically on crimp geometry as the resulting confinement and deformation condition governing contact topology, void formation, and interfacial load transfer behavior under coupled environmental and electrical exposure.

The current work uses systematic isolation of crimp geometry as the main controlling parameter and examines its effects under combined environmental and chemical loads. Consistent environmental and chemical exposure conditions are used not for qualifying products or complying with regulations, but to determine geometrically dependent degradation behavior in a physical stress environment. Electrical conductance and load-carrying capacity were measured through relative changes in resistance and tensile strength, respectively.

This study is not intended for product qualification or compliance, but rather to establish an experimentally transferable framework for assessing early-stage degradation in crimped bonding connections. By incorporating environmental exposure with geometry-dependent crimp configurations, this work identifies trends in coupled electrical and mechanical degradation behaviors. It delineates a transferable framework for understanding geometry-dependent degradation mechanisms beyond a specific cable-terminating implementation, where crimp geometry is not only an assembly parameter, but rather a governing parameter that controls degradation behaviors in exposure environments.

Within this scope, the present study pursues the following four specific research objectives:

1. To experimentally isolate crimp geometry as the primary independent variable governing the coupled electrical–mechanical degradation behavior of ASNE0092 aerospace bonding cables by comparing three representative crimp configurations (Type A, W form; Type B, symmetric; and Type C, oval form) assembled under otherwise identical material and process conditions.
2. To quantify the contribution of crimp geometry relative to environmental exposure parameters (thermal cycling between −55 °C and +70 °C and high-humidity aging at 90–95% RH) on the relative resistance variation ΔR (%) and tensile strength variation ΔT (%) through a replicated full-factorial ANOVA framework at a 95% confidence level.
3. To identify the dominant degradation mechanism under coupled chemical–electrical loading by exposing the three crimp geometries to representative aircraft fluids (Jet A-1, Skydrol, and Type I de-icing fluid) combined with short-circuit current levels of 10 A, 25 A, and 45 A, and to determine whether the geometry-driven response observed under thermal–humidity exposure persists or is superseded under multi-stress conditions.
4. To establish the microstructural and numerical basis of the observed geometry-dependent behavior by correlating SEM-based void fraction and contact continuity analyses with FEM simulations of geometry-dependent terminal deformation, thereby providing a transferable experimental–mechanistic framework for assessing early-stage degradation in crimped bonding connections beyond the specific cable–terminal system investigated here.

## 2. Materials and Methods

### 2.1. Specimen Materials and Assembly

Bonding cables that comply with the standard specification ASNE0092 [15] were used as the test specimens. Nickel-coated bonding cables are widely employed for grounding applications in aerospace engineering. The total cross-sectional area of the copper conductors within the bonding cable is 13 mm^2^. Also, the coating of nickel both inside and outside the conductor gives them thermal stability and resistance against corrosion at temperatures up to 250 °C [15].

Terminal connections consisted of commercially available NSA936020-05N crimp terminals (TE Connectivity, Harrisburg, PA, USA) manufactured from a copper beryllium alloy with a nominal thickness of 1.52 mm and a nickel coating supplied in accordance with Airbus Group SAS/ASD-STAN requirements [16]. This terminal configuration was selected as a representative aerospace bonding interface to ensure material consistency and repeatable crimping conditions throughout the experimental program.

To isolate the effect of crimp geometry on the measured properties, all other parameters, including materials and assembly conditions, were held constant across all experiments. The crimping process followed assembly parameters defined in accordance with the MIL-DTL-83413C standard published in 2012 [17]. This experiment did not aim to prove the qualification of crimps according to the requirements mentioned above, but focused on studying the effect of geometrical modifications on the degradation of the connection system.

All specimens were subjected to visual inspection prior to testing. Specimens were then subjected to 24 h of stabilization in controlled environments at 23 ± 2 °C and 50 ± 10% relative humidity in a controlled laboratory setting. The main material and assembly parameters are presented in Table 1.

A single calibrated crimping tool was used throughout, with all specimens crimped using the same tool and die setup to eliminate variability attributable to tooling or operator technique. All crimping was done in the laboratory under controlled conditions (temperature of 23 ± 2 °C and relative humidity of 50 ± 10%). The crimping force was measured using a calibrated load cell (200 N capacity, Nidec-Shimpo, Glendale Heights, IL, USA).

The objective of the present study was not certification-level qualification or compliance verification of the investigated assemblies, but rather the establishment of repeatable and geometry-controlled deformation conditions for comparative degradation assessment. Accordingly, the procedures and dimensional references defined in MIL-DTL-83413C (2012) were used as process guidance criteria to ensure assembly consistency and reproducibility among the investigated specimens. Following crimping, all specimens were examined using high-resolution optical and metallographic methods to verify deformation morphology, crimp geometry characteristics, and assembly consistency prior to environmental and mechanical testing.

### 2.2. Crimp Geometry Characterization

**Figure 1:**
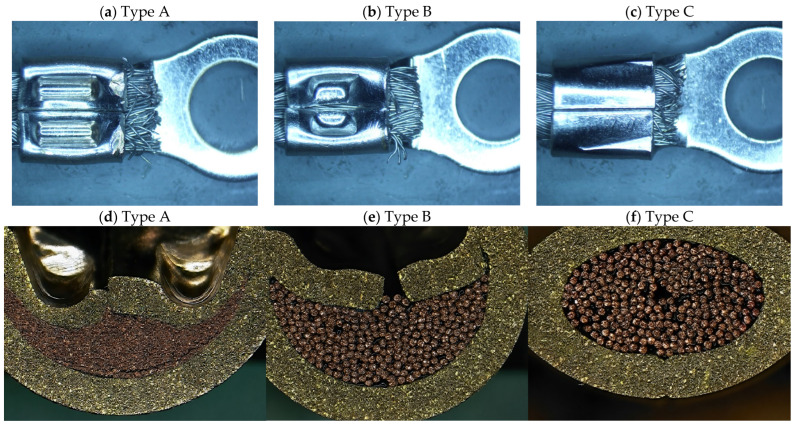
The external morphology of the crimped terminals is shown in (**a**–**c**), while the cross-sectional views obtained after sectioning are presented in (**d**–**f**).

Figure 1 illustrates examples of crimp structures (Type A, Type B, and Type C) with their respective cross-sectional structures. The outer shape of the crimped terminal is illustrated in Figure 1a–c, whereas Figure 1d–f shows the cross-section structure obtained after the sectioning process.

Each of these geometries causes a unique deformation of the terminal, which produces a specific conductor confinement and contact mechanism within the crimp zone. Cross-sectional images acquired using an optical vision measurement system (INSIZE ISD-V250A, INSIZE Co., Ltd., Suzhou, Jiangsu, China) provide further insight into the inner deformation and conductor bundle of each crimp structure.

The crimp geometries considered were selected to represent different deformation and confinement modes that may arise during actual crimping and assembly processes. The Type A geometry corresponds to high compression, approaching the reference crimp mode. Types B and C correspond to decreasing compression and correspond to non-uniform deformation and insufficient confinement, which could be caused by variations in the assembly process or improper crimping. It should be noted that these geometries were not intended to represent three equally qualified aerospace crimping standards. Rather, these geometries represent controlled changes in the level of confinement quality aimed at studying the effects of deterioration dependent on geometry due to the combined effect of the environment, chemistry, electricity, and mechanics. In this regard, Type C represents a deliberately reduced compaction level, intended to replicate a suboptimal crimp geometry.

The relatively compact strand arrangement in the Type A cross-section shown in Figure 1d reflects the higher compaction and deformation in this crimp geometry. Due to the lower void fraction and higher confinement, the strands in this specimen have made more interfacial contact, resulting in their apparent merging at the boundaries in the metallographic image.

**Figure 2:**
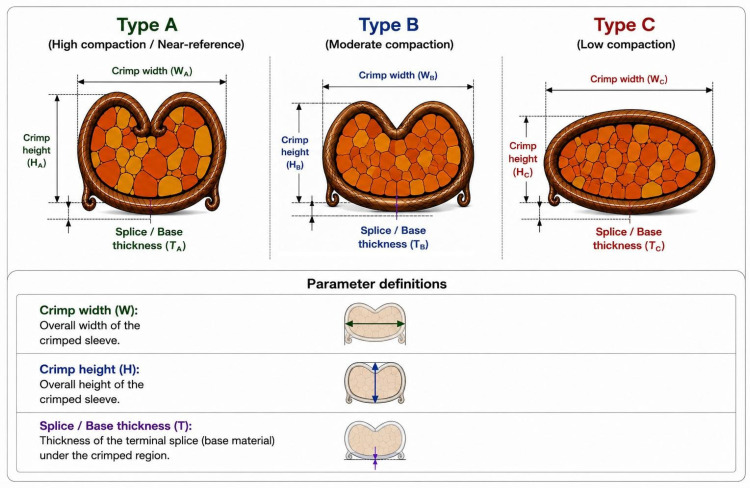
Schematic representation of the three crimp cross-section geometries with different compaction levels: Type A, Type B, and Type C.

The dimensions of the crimp geometries examined, together with the relevant parameters used to characterize the deformation profile, are shown schematically in Figure 2. These dimensions include crimp width (W), crimp height (H), and splice/base thickness (T). These dimensions are directly related to the degree of conductor confinement, deformation profile, and contact formation within the crimped section. Variations in these dimensions imply different levels of compaction and confinement achieved during crimping and assembling. The overall crimp geometry is consistent with the established deformation characteristics reported for such crimp structures [18].

Some geometric features can be used to define the crimp properties. The selected geometric parameters in this research were those that influence mechanical confinement and behavior under deformation. This includes crimp height, crimp width, and splice/base thickness. Crimp height and crimp width affect the global deformation behavior, while the other geometric feature affects the local load transfer.

In the current research, crimp height and crimp width were defined using a digital caliper. Calibration should be performed periodically to guarantee accurate results. Splice/base thickness is an important factor in load transfer behavior. It is calculated through SEM-assisted measurement after specimen preparation. Five readings were obtained from the base area to the nearest conductor voids perpendicular to the base area.

Each crimp geometry was assessed using three separate cross sections. In each cross-section, five readings for thickness were recorded from the base towards the nearest voids in the conductor within a direction normal to the base plane. The recorded values are reported as mean ± standard deviation. The corresponding geometric parameters measured from the cross-sectional profiles are summarized in Table 2.

### 2.3. Electrical Resistance Measurement and Tensile Testing

**Figure 3:**
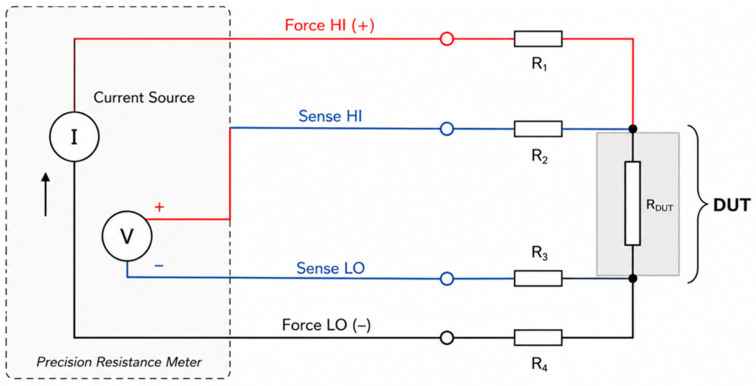
Schematic representation of a four-wire (Kelvin) resistance measuring circuit.

Electrical behavior was characterized through low-resistance measurements using the four-wire (Kelvin) connection method, which eliminates errors attributable to lead resistance [19]. Specimen resistance was determined in milliohms using a micro-ohmmeter. Measurements were taken both before and after environmental exposure. In each measurement session, five readings were averaged to minimize measurement uncertainty. Electric contact was made directly at specific areas. Figure 3 shows the schematic representation of a four-wire (Kelvin) resistance measuring circuit.

Post-exposure resistance variation was evaluated using both absolute change (R_1_ − R_0_) and relative variation, which is defined as:

(1) $$\Delta R\%=\frac{R_{1}-R_{0}}{R_{0}}\times 100$$

**Figure 4:**
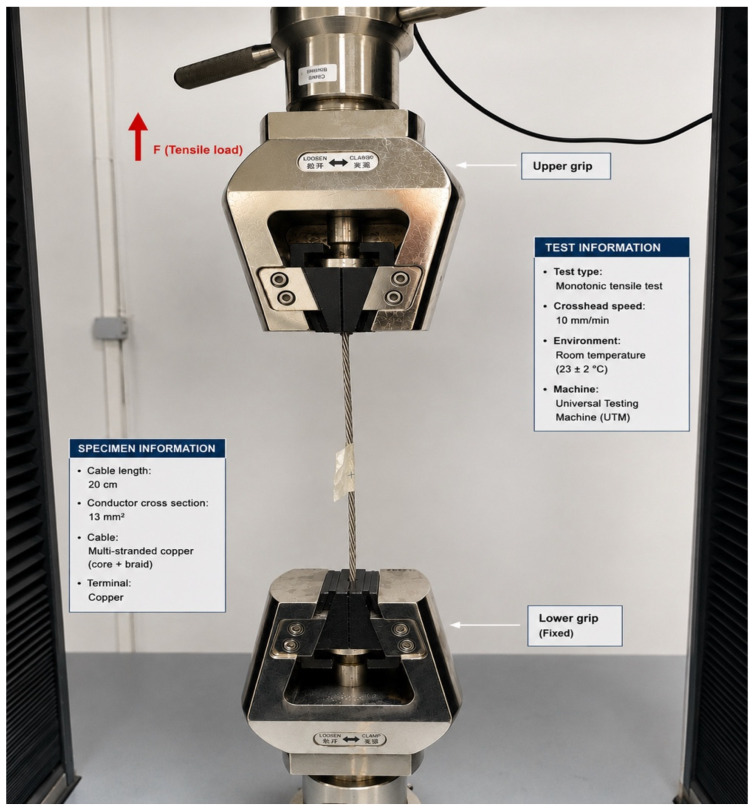
Test setup used to perform tensile testing on crimp-bonded cable samples.

The mechanical performance of the crimped joints was quantified by tensile testing following procedures adapted from MIL-STD-1353C (2014) [20]. The tensile tests were performed in displacement-controlled mode using a universal tensile testing machine (TIME Group Inc., Beijing, China). During the test, one end of each specimen was secured with a mechanical jaw grip, while the other end was held with an eye pin grip that is coaxial with the loading axis of the testing machine, as illustrated in Figure 4. This fixture combination was selected to avoid bending, eccentricity, rotational motion, and slippage during testing. Tensile loads were applied axially at a constant crosshead speed of 10 mm/min up to the breaking point. The ultimate tensile load was taken as the measure of mechanical performance. Three specimens per condition (*n* = 3) were subjected to tensile loading and gripping under the same testing conditions. The load–displacement curves presented below correspond to the mean values obtained from the test specimens, while statistical analysis was carried out based on individual measurements.

After each tensile test run, the specimens were analyzed using both visual inspection and stereo microscope observation to classify the type of failure observed. Failure modes were pre-defined based on MIL-STD-1353C (2014) and the same categories described in [5,6] as:

(a) Conductor wire fracture (CWF): Failure caused by tensile fracturing of conductor wires outside the crimp barrel, with wires still retained in the terminal. This failure mode indicates full mechanical engagement between the conductor and terminal; accordingly, the recorded force represents the tensile strength of the conductor.
(b) Mixed mode failure (MMF): Partial fracture of the conductor together with lateral disengagement of the wires from the crimp barrel. This mode indicates only partial mechanical engagement, with the conductor wires transferring loads to the terminal under conditions of partial deformation or slippage.
(c) Conductor pull-out/slip (CPO): Complete pulling out or slippage of the conductor wires without any fracture occurring. In this case, the recorded force does not represent the tensile strength of the conductor material, but rather the engagement strength of the conductor bundle within the crimp barrel.

Accordingly, identification of the failure mode plays a crucial role in interpreting ΔT (%) measurements, as conductor wire fracture suggests degradation of the conductor material and/or its interface, while conductor pull-out suggests poor mechanical engagement of the crimped joint.

Post-exposure tensile strength variation was evaluated using both absolute change (T_1_ − T_0_) and relative variation, which is defined as:

(2) $$\Delta T\%=\frac{T_{1}-T_{0}}{T_{0}}\times 100$$

This parameter was used as a comparative indicator of geometry-dependent mechanical degradation.

### 2.4. Environmental Exposure and Fluid Immersion Tests

To evaluate the performance of crimped bonding joints under degrading conditions, specimens were subjected to controlled temperature, humidity, and chemical exposure conditions representative of aerospace operational environments. The temperatures and humidity levels used in the tests were selected based on the exposures defined by RTCA DO-160G (2010) and ISO 16750-4 (2023) [21,22].

**Figure 5:**
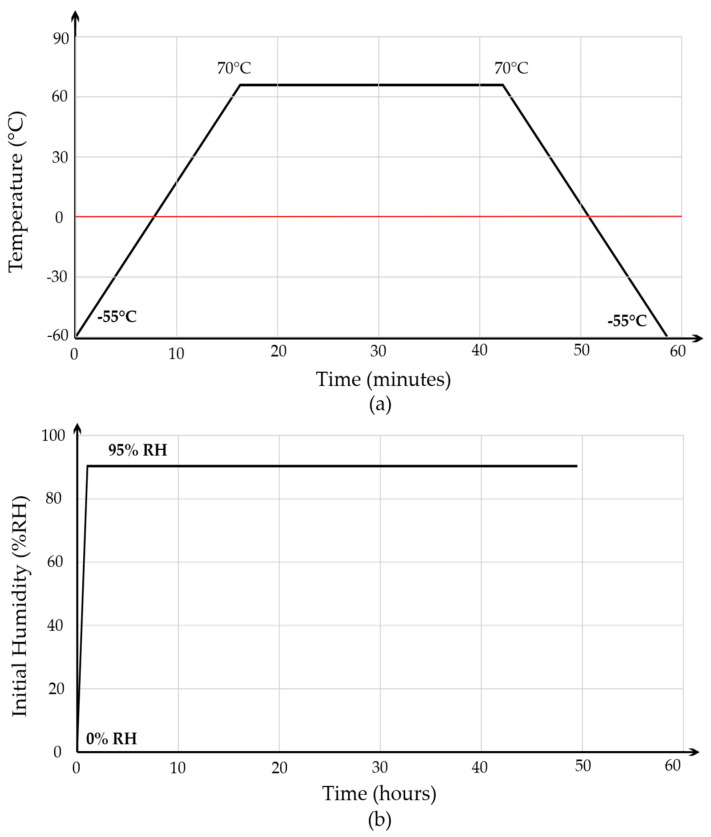
Environmental exposure profiles applied in this study: (**a**) temperature cycling between −55 °C and +70 °C with stabilization periods at each temperature level; (**b**) the relative humidity profile (90–95% RH) used during environmental aging tests.

All temperature cycles were carefully controlled regarding temperature change rates between −55 °C and +70 °C, as shown in Figure 5. Starting from ambient room temperature (approximately 25 °C), specimens were cooled to −55 °C over approximately 20–25 min and held at this temperature for 30 min to achieve thermal stabilization. Next, the temperature inside the chamber was raised from −55 °C up to +70 °C within 15–20 min, and specimens were stabilized for another 30 min inside the chamber at +70 °C.

Upon completion of each heating phase, the specimen’s temperature inside the chamber dropped back to −55 °C, and the process was repeated continuously throughout the entire duration of the tests. Based on the total exposure time, several thermal cycles were carried out consecutively with identical chamber conditions set. In the current research, exposure times were evaluated according to four different scenarios, 8 h, 16 h, 24 h, and 48 h, which were regarded as different time points of degradation processes.

Following thermal cycling exposure, specimens were exposed to humid air in the same environmental chamber, with the aid of a humidity control system based on an industrial chiller. The chamber temperature was set to a range of 30–33 °C, while the relative humidity inside the chamber reached 90–95% RH, as shown in Figure 5. To achieve the proper temperature and humidity level inside the chamber, 1 h was needed, after which stable humidity exposure began.

Fluid immersion tests were conducted using representative aircraft operational fluids, which include Jet A-1, Skydrol hydraulic fluid, and Type I de-icing fluid. The test procedure followed RTCA DO-160G (2010) 11.4.2 “Fluid susceptibility—Immersion.” All samples were subjected to total immersion of the testing fluid for 24 h inside a temperature-controlled chamber. Immersion temperatures for each fluid were assigned in accordance with Table 11-1 of RTCA DO-160G (2010). For the immersion testing procedure, static immersion test conditions were applied, with each specimen fully submerged throughout the exposure period [21,22].

After immersion, surface droplets were manually wiped away without any cleaning solvents to maintain the conditions of the remaining surface fluid and to create a conservative worst-case service fluid scenario. Prior to electrical loading, the samples were kept under control lab conditions for 2 h.

Electrical loading was applied immediately after fluid exposure using a Chroma 19572 Ground Bond Tester (Chroma ATE Inc., Taoyuan City, Taiwan). Current loads of 10 A, 25 A, and 45 A were supplied for 60 s. The current was turned off, and then the sample was allowed to reach a stable temperature for 1 min, 3 min, and 5 min in response to the 10 A, 25 A, and 45 A loads, respectively. Electrical resistance measurements were then taken. Five sequential measurements were averaged in each case. No thermal control of electrical loading took place. This test phase was conducted exclusively to assess the effect of electrical stress within the scope of this study.

Fluid exposure and electrical loading were conducted as sequential steps within a single experimental protocol. The fluid exposure phase included sequential steps of immersion, stabilization, electrical loading, and post-electric resistance testing in controlled lab conditions. This approach enabled the examination of the interaction between geometry, fluid exposure types, and electrical loading conditions.

### 2.5. Scanning Electron Microscopy (SEM)

To elucidate the mechanisms governing geometry-dependent electrical and mechanical performance, Scanning Electron Microscopy (SEM) analyses were performed on the crimp joint. The aim of the SEM study was to examine the conductor continuity, voids present within the joint, and the nature of deformations experienced at the conductor–crimp interface—all of which affect the electrical and mechanical properties of the joint.

With the above in mind, crimp terminal samples were cut using WEDM. The reason behind using WEDM for cutting was that, unlike other methods of cutting, there are no deformations induced through the cutting process. In addition, no mechanical polishing or etching was performed to preserve the native state of the crimp interface.

Although localized thermal effects are inherently associated with the WEDM process, the low-energy precision sectioning conditions used in the present study did not produce observable remelting, surface smearing, or morphological distortion within the analyzed crimp-contact regions. Therefore, the method was considered suitable for comparative metallographic and void fraction evaluation of the investigated crimp geometries.

Images were obtained using a Thermo Scientific Phenom XL G3 Desktop Scanning Electron Microscope (Thermo Fisher Scientific, Waltham, MA, USA) at 10 kV with magnifications ranging from 67× to 90×, based on the zone of interest. Low magnification images were used to evaluate the distribution of the voids in the crimp zone, while high magnification images were used to determine conductor-to-conductor contact behaviors. The SEM images were analyzed using PRECiV image analysis software (v2.2), where voids were measured by grayscale segmentation.

Three SEM micrographs of the crimped conductor-connector area were examined for each specimen condition under the same magnification levels to allow for comparable analyses between the geometries. The analyses were restricted to the mechanically compressed crimp section that includes the conductor assembly and connector assembly. Void area fraction was quantified by threshold segmentation applied to grayscale micrographs using PRECiV software (v2.2). Threshold values were set manually and held constant across all specimens to ensure consistent void detection across the different crimp geometries. Key SEM parameters are shown in Table 3 below.

### 2.6. Finite Element Modeling (FEM)

The finite element modeling (FEM) technique has been employed to study geometry-dependent deformation characteristics of terminals in the crimping operation. The FEM analysis was used strictly as a qualitative tool to interpret deformation trends observed in the experimental results.

The simulations were conducted using the OptiStruct solver within the Altair HyperWorks (v2026) environment under a three-dimensional nonlinear contact framework. In order to ensure numerical stability, the FEM model was discretized using structured hexahedral solid elements. Both the crimping jaws and the terminal geometry were modeled using solid mesh structures. Linear elastic steel material properties were assigned to the crimping jaws, whereas the terminal material was defined using an elastoplastic material model to represent the permanent deformation occurring during the crimping process. The steel material properties used in the analysis are summarized in Table 4. Surface-to-surface contact interactions between the tooling and terminal surfaces were defined using a penalty-based contact formulation with a constant friction coefficient representative of metal-forming conditions.

The model did not explicitly represent the multi-strand copper conductor. Consequently, it could not account for individual strand deformation, inter-strand friction, conductor-induced pressure on the terminal, or the mechanical locking effect. Owing to the failure to include the conductor, it is also difficult for the model to replicate the exact deformation of the terminals when there is crimping. As a result, finite element modeling was not used to simulate electrical resistance behavior.

In addition to that, the numerical analysis model did not include a specific criterion for the initiation, propagation, and failure of the material. Instead, FEM results were used qualitatively to identify trends in deformation, confinement, and lateral expansion as a function of crimp geometry. As such, the final crimp geometry was considered only as another measure of deformation trends rather than a failure criterion.

The FEM model was developed based on the NSA936020-05N crimp terminal geometry. To ensure that material behavior is accurately simulated during the process of FEM simulation, not only the elastic properties but also the true stress–true strain relationship characterizing the plasticity regime of the material were considered. Mechanical properties and the true stress–true strain relationship used for the simulation are shown in Table 5 and Table 6, respectively.

Three-dimensional finite element models representing Type A, Type B, and Type C crimping configurations were constructed based on experimentally observed crimp geometries. To isolate the deformation characteristics associated with crimp formation and to reduce computational complexity, the numerical model included only the crimping jaws and the terminal component, while the conductor was not explicitly modeled.

**Figure 6:**
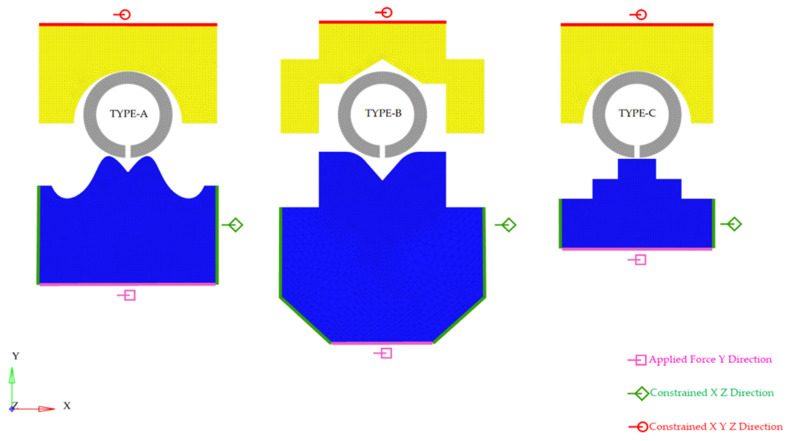
Boundary conditions and loading configuration applied during the crimping simulation for the three different jaw geometries (Type A, Type B, and Type C). The upper jaw is constrained in the X and Z directions and subjected to a 200 N force in the Y direction; the lower jaw is fully constrained in all three directions.

The lower jaw was fully constrained in the X, Y, and Z directions, whereas the upper jaw was constrained in the X and Z directions. To represent the mechanical forming action during the crimping process, a force of 200 N was applied to the upper jaw in the Y direction. This loading and constraint configuration can be observed in Figure 6.

**Figure 7:**
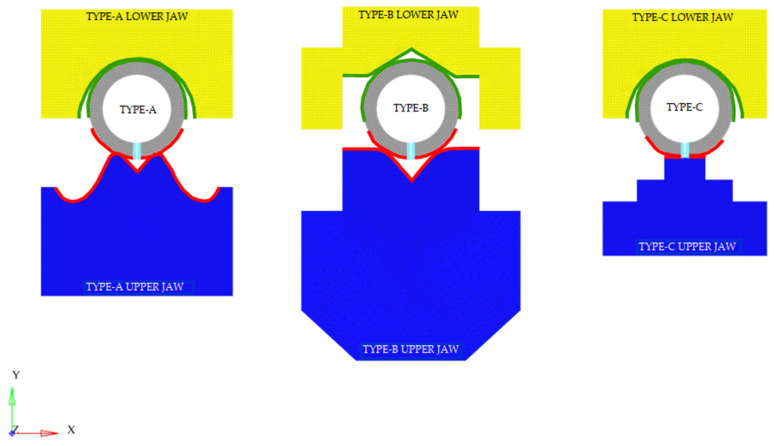
Contact surfaces defined in the FEM model for Type A, B, and C crimping geometries.

Surface-to-surface contact interactions with friction were established at the interface between the jaws and the terminal surfaces. A static friction coefficient of 0.36 was assumed for the contact interface between the copper terminal and the steel jaws. The contact surfaces are illustrated in Figure 7. The interaction between the inner surface of the terminal and the conductor was not modeled. Only the terminal deformation was considered. Since the conductor was excluded from the model, conductor–terminal and conductor–conductor contact mechanics were not captured, and the FEM results are therefore limited to describing terminal deformation behavior.

Simulation outputs evaluated in this study consisted of displacement distributions within the terminal, which were used to interpret geometry-dependent deformation behavior arising during the crimping process.

### 2.7. Statistical Analysis (ANOVA)

Statistical analysis of experimental data was performed using a full factorial analysis of variance (ANOVA). Three independent specimens were used (*n* = 3) per experimental condition, with each replicate considered independently during any statistical calculations. Values reported in tables represent the means of three independent measurements. Analysis of variance was performed with raw replicates rather than means to include between-condition variance as part of the model’s error term. Dispersion measures (standard deviation) for all conditions are given in the tables and figures to provide information about specimen-to-specimen repeatability. While reported values correspond to averages of these measurements, statistical analysis was done using the corresponding data sets to preserve experimental variability. Prior to the interpretation of ANOVA results, basic statistical assumptions were checked. The normality of residuals was assessed using the Shapiro–Wilk test. Despite significant departures from normality (*p* < 0.001, W = 0.893 for ΔR (%); *p* < 0.001, W = 0.935 for ΔT (%)), factorial ANOVA is relatively robust to the non-normality of residuals when there is equal cell size in a balanced design, which is valid for the present experiment. The homogeneity of variance was assessed by Levene’s test; homogeneity was confirmed for ΔR (%) (F = 2.69, *p* = 0.074), while the homogeneity of variance of ΔT (%) was borderline (F = 3.12, *p* = 0.050), which is admissible for a robust ANOVA analysis. Temporal autocorrelation was examined using the Durbin–Watson statistic calculated based on an ordered sequence of residuals. Low DW statistics values for both ΔR (DW = 0.31) and ΔT (DW = 0.09) indicated systematic ordering of the runs according to their factor levels, but not autocorrelation since data were not gathered as a time series. Replicate independence was ensured by conducting measurements on three physically independent specimens per condition. The significance level was set at 95% (*p* < 0.05).

## 3. Results and Discussion

### 3.1. Mechanical Performance and Failure Characteristics

The mechanical response of the crimped connections was evaluated based on tensile load–displacement behavior and post-failure characteristics.

**Figure 8:**
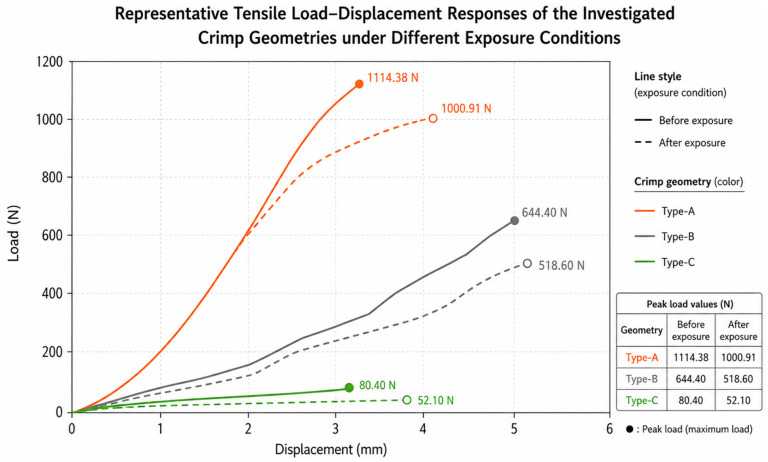
Representative tensile load–displacement responses of the investigated crimp geometries (Type A, Type B, and Type C) before and after environmental exposure.

**Figure 9:**
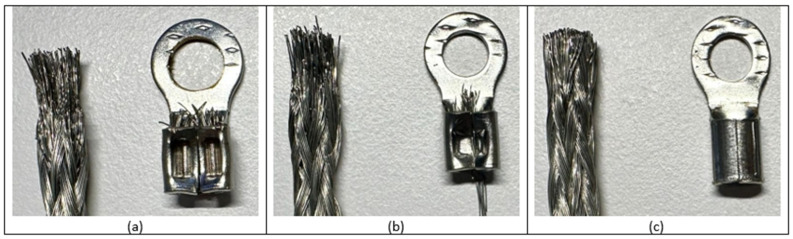
Fracture patterns observed in Type A, Type B, and Type C crimping specimens after 48 h of temperature/humidity exposure: (**a**) Type A, (**b**) Type B, and (**c**) Type C.

The tensile load–displacement curves presented in Figure 8 provide insight into the mechanical behavior of the three crimp configurations under exposure conditions. The mechanical behavior of these crimp configurations is greatly influenced by their deformability characteristics and contact density, as revealed in the SEM images of Section 3.2. The Type A geometry shows the best mechanical response because of symmetric deformability and efficient clamping. The Type C geometry shows poor mechanical response due to the low effective contact area and stress concentration. These findings are consistent with the statistical results and are further supported by the type of fracture observed after the tensile test. Specifically, complete conductor fracture was absent, with strand pull-out being the predominant failure mode. This observation indicates poor mechanical interlockage and poor confinement caused by the small plastic deformation of the wire strands while being subjected to the crimping process. Notably, the failure modes observed here are consistent with the reduced contact continuity and higher void fraction observed in the SEM observations. The fracture behavior observed in the specimens is presented in Figure 9.

The above classification was based on visual observations done using optical and stereomicroscopy for all samples tested (*n* = 3 per specimen type). For Type C, none of the specimens experienced conductor breakage during testing under the test conditions used. The applied loads were constant, and the pull-out force limit is determined by the interaction between friction and interlocking mechanisms occurring within the oval crimp barrel housing. The absolute T0 of the Type C specimen type is 73.8 N and constitutes about 6.6% of the absolute value of the Type A specimen, which is indicative of the pull-out dominating the behavior of the former.

Figure 8 should be considered in relation to Table 7. For specimen Type A, the occurrence of conductor wire fracture (CWF) outside the crimp barrel indicates that the W form geometry produces sufficient plastic interlock for the conductor bundle to engage properly into the crimp terminal. Therefore, the load applied, ranging from 1009 to 1053 N depending on the exposure period, matches the ultimate tensile strength of the engaged conductor. Conversely, the Type B specimen shows a mixed-mode failure (MMF) characterized by partial rupture and visible displacements of the conductor bundle from the crimp barrel, implying that only a part of the conductor strands transmits loads through plastic interlock. This contrasts with the Type C specimen, which is characterized by conductor pull-out (CPO), where the whole conductor bundle is pulled out from the crimp barrel without strand fracture.

The extremely low absolute tensile values recorded for Type C after exposure (T_1_ = 50–55.8 N cannot be attributed to changes in conductor tensile strength since no strand fracture occurred. Rather, the results indicate limited mechanical engagement arising from the crimp’s inability to achieve effective interlock due to inadequate plastic confinement (14.25% void fraction, hence insufficient friction and interlock forces. The influence of chemical exposure type and crimp geometry on tensile strength degradation is presented in Table 8. In summary, the values indicated in Type C ΔT (%) can be attributed to two factors: (i) the as-crimped geometrical limitation in terms of mechanical engagement; (ii) the additional deterioration of the engagement caused by environmental ingress in the surface contact region (insufficient confinement potential in FEM, Section 3.2 and Section 3.3).

As can be seen from the experimental results, Skydrol produced the greatest ΔR (%) increase relative to the other fluids and current levels across all crimp geometries, followed by intermediate values obtained for Type I de-icing fluid, whereas the least ΔR increment was detected upon exposure to Jet A-1. These results correlate well with the established physicochemical features of the considered fluids. Skydrol is the phosphate ester-based hydraulic fluid possessing the highest degree of polarity and low surface tension, resulting in enhanced surface wetting and possible interaction with the metallic oxide layer [25,26]. Jet A-1 consists of nonpolar aliphatic hydrocarbons demonstrating low surface reactivity towards nickel or copper interfaces [27]. Type I de-icing fluid includes a considerable amount of propylene glycol characterized by its hygroscopic properties [28]. It must be acknowledged, however, that this study did not include surface spectroscopic analysis to establish the presence of chemicals involved in the reaction. Hence, the fluid ordering proposed here can be considered experimentally derived but is not based on the actual surface identification of reaction products or corrosion-related phenomena. Accordingly, the fluid-dependent degradation trends reported above should be regarded as physicochemical correlations between exposure and measured parameters rather than direct verification of the possible mechanism of interfacial interaction. Therefore, the effects of chemical exposure on electrical and mechanical properties of crimps can be analyzed on the grounds of observed data and trends rather than elemental analysis. More accurate conclusions about the mechanisms of interaction between metals and particular fluids can be formulated once the dedicated surface analysis is conducted. It should also be noted that the selected fluids serve different operational functions within aircraft systems; however, they represent various classes of aerospace service fluids.

**Figure 10:**
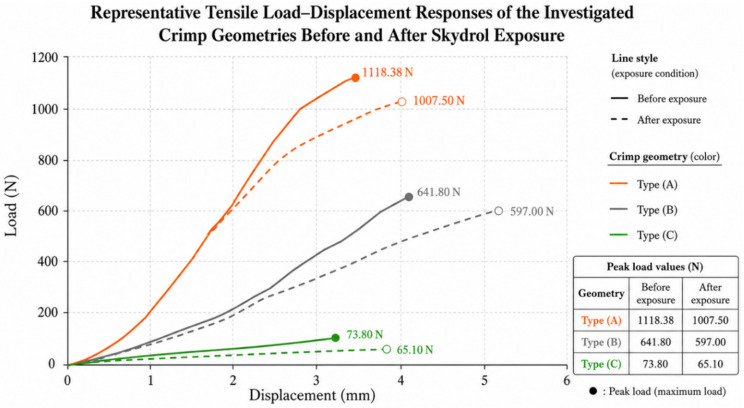
Representative tensile load–displacement responses of the investigated crimp geometries before and after Skydrol exposure.

The load–displacement graph corresponding to Figure 10 shows the substantial degradation of mechanical behavior due to the influence of chemicals, with significant changes for non-optimal crimp structures (Types B and C). While the Type A structure retains its load-bearing behavior, the tensile strength in the Type B and, even more significantly, the Type C structures, is substantially decreased. This latter effect is associated with the changes in interfacial behavior at the crimp area, where possible residual effects from the chemical fluids can affect surface behavior, leading to the formation of an interfacial layer and instability in the conductors’ interconnectivity, especially when void distribution and low compaction are present. Such a phenomenon may not only cause mechanical degradation but also contribute to the disruption of conductive pathways and higher resistance due to simultaneous chemical and electrical influence. The results show that the effect of chemical fluid exposure depends on the crimp geometry.

The connection between crimp geometry, stress–displacement behavior, and microstructure provides important insights into the mechanics of performance seen in the experiments performed. The tensile stress–displacement plots illustrated in Figure 8 and Figure 10 show that the mechanical behavior of the connector is not just a material characteristic but is directly related to the contact geometry and the patterns of plastic deformation formed during the crimping process. Stress–displacement behavior can be explained based on three different inter-related criteria: (1) initial contact area and load transfer mechanism; (2) confinement quality and plastic deformation of crimping region; and (3) connectivity or fragmentation of conductive networks created by crimping.

Type A is characterized by the steepest initial slope and the highest ultimate tensile load, ranging from 1009 to 1053 N, with minimal degradation (ΔT% = 5.8–9.4%) despite different exposure conditions. The best mechanical properties of Type A are related to its minimal void fraction (0.46% in Table 9). Significant plastic deformation leads to conductor-to-conductor contacts, where the boundaries of strands disappear and a percolated conductive network is created with excellent load transfer properties. Since plastic deformation occurs uniformly across the crimp region, tensile load is divided into multiple conductive paths, and the stress–displacement plot maintains a high slope until fracture of the conductor wires. In such cases, the material response is ductile, and the ultimate tensile strength is determined by the properties of the conductor rather than by the joint interface.

On the other hand, Type B crimp geometry demonstrates an average stress–displacement behavior (Figure 8). The values of ultimate tensile loads range between 518 and 642 N with a gradual increase in deterioration (ΔT% growing from 8.3% to 19.6%). Type B shows moderate void volume (7.98% in Table 9) with partial conductor-to-conductor contact (Table 10). Partial plastic deformation means that boundaries between strands are preserved, leading to discontinuous conductive networks, where voids are present. Discontinuity means fewer options for load transfer and, therefore, a lower slope of the stress–displacement curve compared to Type A geometry.

Finally, Type C crimp geometry demonstrates the least desirable stress–displacement behavior. The ultimate load ranges from 50 to 80 N, which corresponds to 6.6% of the ultimate load of Type A (Table 7 and Figure 8). Failure occurs exclusively by conductor pull-out (CPO) rather than in conductor wire fracture (CWF) mode. This indicates that the problem of Type C crimp geometry is a lack of engagement rather than weakening of the material strength. Thus, the stress–displacement plot for Type C has a very small initial slope, and the maximum load happens in the elastic part of the curve. Further displacement occurs due to friction-limited sliding. Therefore, the absolute value of the initial load (T0 = 73.8 N) reflects only the friction engagement of loosely connected strands and not the actual tensile strength of copper itself. As a result, despite the very poor mechanical performance of Type C connectors, ΔT% is minimal (3.5–5.3%) compared to other types of geometries. The foregoing analysis confirms that mechanical performance is entirely determined by the microstructural state established during crimping. Plastic deformation depends on geometry and results in either a continuous or a disconnected conductive network, depending on the volume fraction of voids.

**Figure 11:**
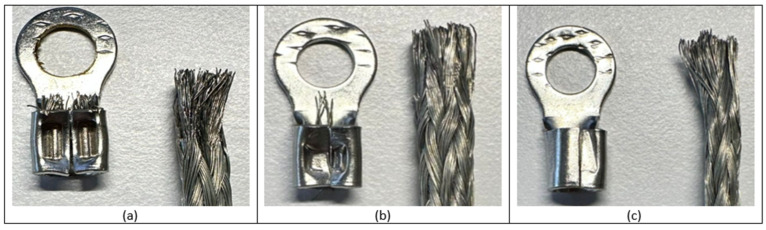
Fracture patterns observed in Type A, Type B, and Type C crimping specimens after exposure to Skydrol hydraulic chemical exposure: (**a**) Type A, (**b**) Type B, and (**c**) Type C.

Failure mechanisms for Types A–C in the post-exposure condition, as shown in Figure 11, confirm the failure mode classification discussed in Table 7 and reveal the chemical effect influence on the failure of each individual type. Regarding Type A (Figure 11a), the conductor wire fracture (CWF) mechanism remains unchanged after Skydrol application, showing only slight changes in the orientation of the fracture plane compared to the original unexposed specimen; therefore, the W form geometry still allows enough engagement to exert a tension force on the conductor. Type B (Figure 11b) remains in the MMF group; however, the share of pulled-out wires becomes higher than the fractured wires, reflecting a loss of partial engagement due to penetration of the fluid into the engagement zone by the capillary effect. The same applies to Type C (Figure 11c), which is characterized by a complete pull-out (CPO) mechanism in precisely the same way as in the original unexposed specimen, while the recovered conductor has completely wetted strands in the whole engagement area with the former barrel. Concerning the failure modes, there is no effect from chemicals; the modes work at decreased load levels, especially Type B, which is marginal from the very beginning. This also explains why Type C exhibits relatively low ΔT (%) values, about 3.5–5.3%, in Table 7 and Table 15: as this conductor already works in pull-out mode, the effect of chemical degradation plays a secondary role to the geometric parameters.

Failure mode dependence on geometry is determined by the initial contact area established during crimping, which in turn controls the stress distribution pattern and load-carrying capabilities of the joints. Mechanistically speaking, the findings presented herein can be compared to the comprehensive work recently done by Liu et al., which concerns the electrical failure of contact terminals in automotive connectors under the combined temperature, vibration, and pull/plug loading conditions [29]. Their results demonstrated that reduced contact area promotes local stress concentration and micro-crack formation at the terminal–conductor interface, which is consistent with the contact fragmentation observed in Type C specimens with inadequate compaction (7.98% void fraction in Type B; 14.25% in Type C). The contact topology dependence of electrical and mechanical parameters has also been confirmed experimentally through numerical studies conducted by Dankat and Dumitran [30]. Specifically, in their paper, they provided quantitative evidence that the effective contact area, determined by plastic deformation, defines both electric conductivity and mechanical performance of low-current electrical contacts. The correlation between void fraction metrics and failure mode changes can be explained by nonlinear mechanical deterioration resulting from insufficient growth of contact area under increasing load. Additionally, Yang et al. demonstrated that the initial contact state—defined by crimping-induced plastic deformation and the resulting surface roughness—governs the subsequent evolution of contact behavior under mechanical and thermal loading in current-carrying frictional contacts [31]. Taken together, these independent studies provide additional evidence to the hypothesis that the failure mode dependencies discussed above are general principles of contact mechanics applicable to crimped electrical contacts regardless of applications.

### 3.2. Scanning Electron Microscopy (SEM) Analysis

**Figure 12:**
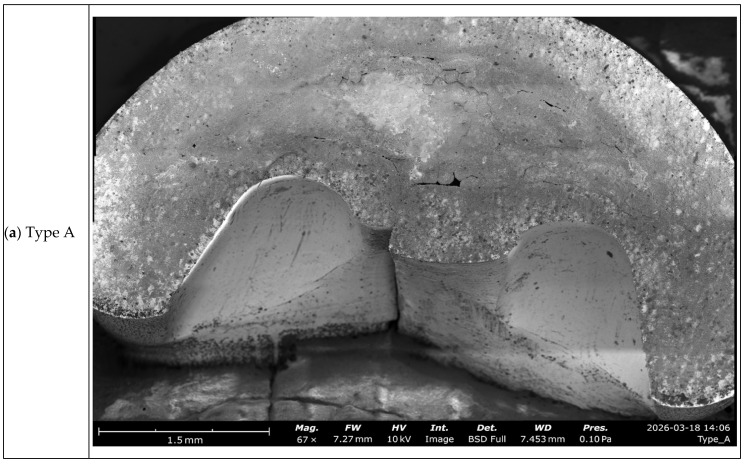

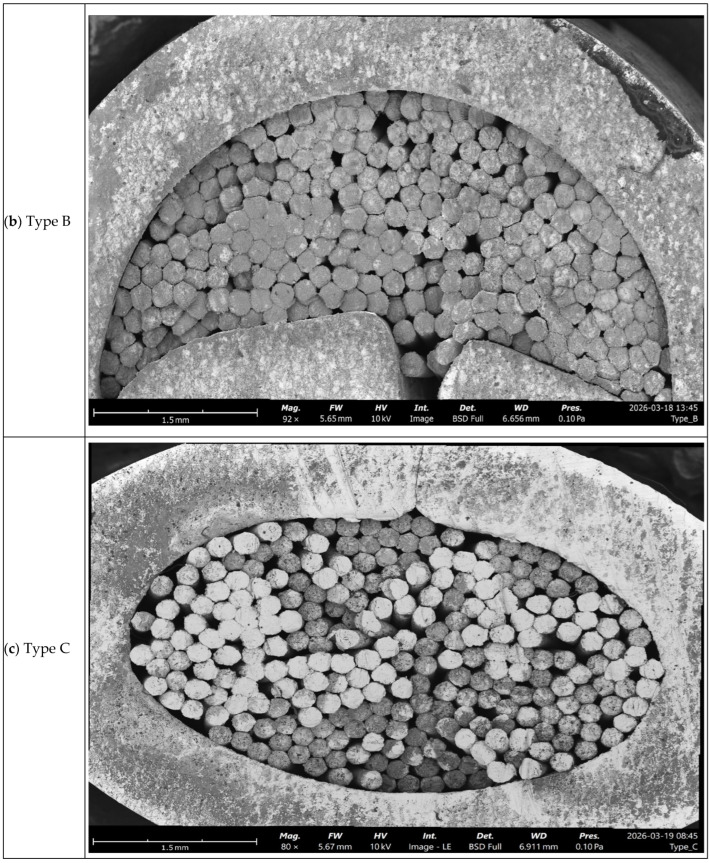
SEM micrographs of the crimp interface for different crimp geometries: (**a**) Type A, (**b**) Type B, and (**c**) Type C. The images illustrate differences in conductor deformation, contact continuity, and void distribution.

SEM analysis was conducted to provide microstructural evidence supporting the geometry-dependent deformation and contact behavior identified in the experimental and numerical analyses. Representative SEM micrographs of the investigated crimp geometries are presented in Figure 12.

The Type A configuration has a very compacted conductor structure, along with significant plastic deformation of each strand. The strands are densely packed, with minimal interstitial gaps, producing a nearly continuous contact interface. The Type B configuration has a moderately compacted conductor structure, where each strand has a more circular shape. In addition, there are distributed voids present in the crimp region. The Type C configuration has the least compacted structure, where there is limited plastic deformation of each strand. Moreover, there are significant voids present between each of the adjacent conductors, resulting in a discontinuous type of interface.

**Figure 13:**
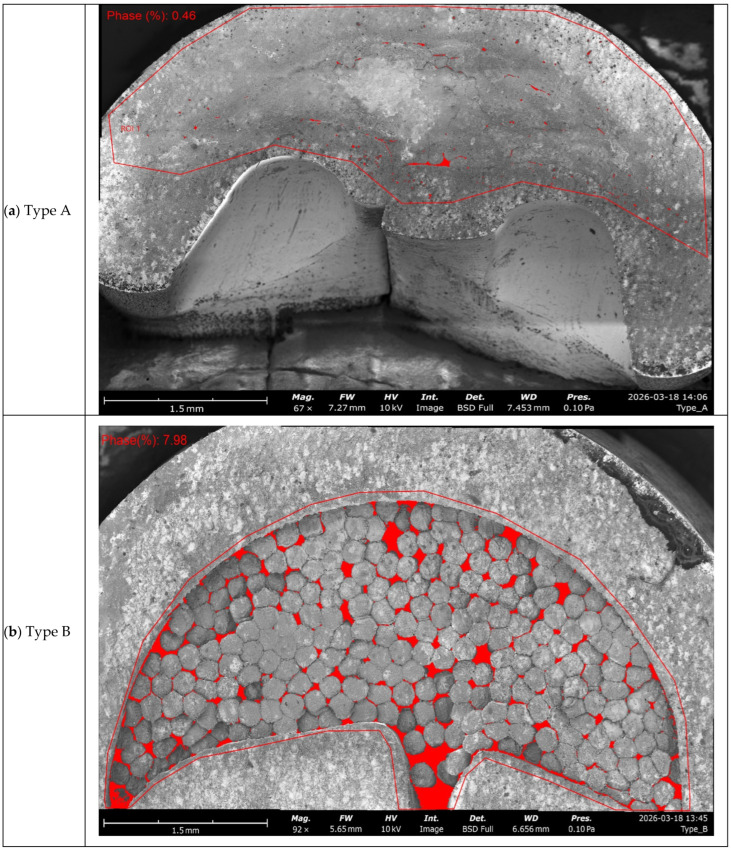

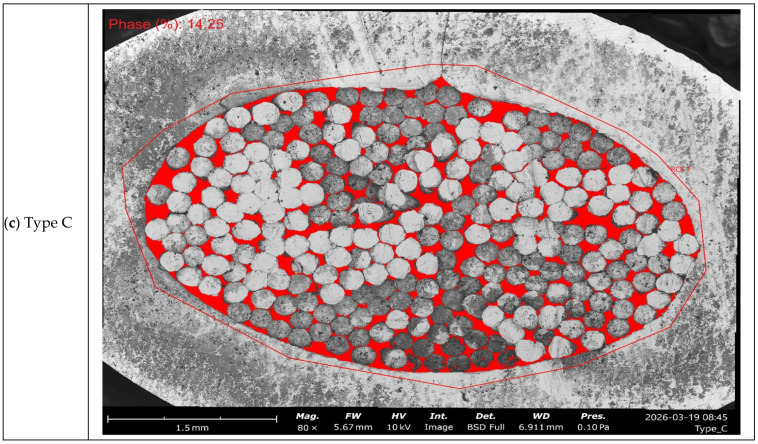
SEM-based void fraction analysis of the crimp interfaces for different crimp geometries: (**a**) Type A, (**b**) Type B, and (**c**) Type C. Void regions are red highlighted, and the corresponding void fractions indicate a clear increase in internal gaps with decreasing crimp compaction.

Image-based analysis was performed to quantify the void fraction present in the crimp region, as shown in Figure 13. The results are presented in Table 9.

The result for the Type A configuration is a minimum void fraction of 0.46%, implying nearly full compaction of the connection. By contrast, the void fraction increases substantially to 7.98% for Type B and 14.25% for Type C, reflecting a significant reduction in effective contact area with decreasing crimp compaction. The reduced contact area limits the number of available conductive paths, thereby increasing electrical resistance. In terms of failure mode, while Types A and B fail through conductor fracture, Type C fails by conductor pull-out owing to low mechanical interlock strength, i.e., the friction between conductors. Such a difference in the mechanism of failure is consistent with what one would expect physically and contact mechanics-wise: the 14.25% void fraction for the Type C connection (refer to Table 9) decreases the normal force on the conductor–terminal interface to the extent that friction becomes ineffective in transferring load. SEM inspection of the specimen’s post-test reveals geometrically intact strands of Type C after the test, indicating that the applied load did not exceed the tensile strength of the conductor strands in a way typical of crimped-wire failure and multiple-strand fracture types of failure mechanisms. Therefore, the lower tensile values obtained for the Type C specimen correspond to a geometry-induced transition in failure modes, in accordance with MIL-DTL-83413C principles of crimped-wire joint design.

**Figure 14:**
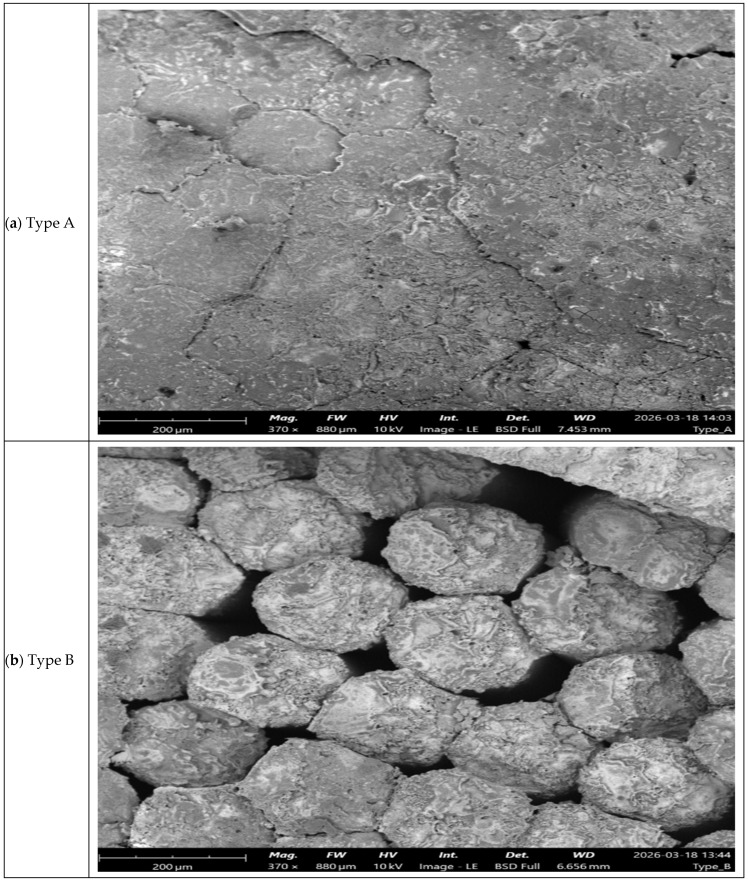

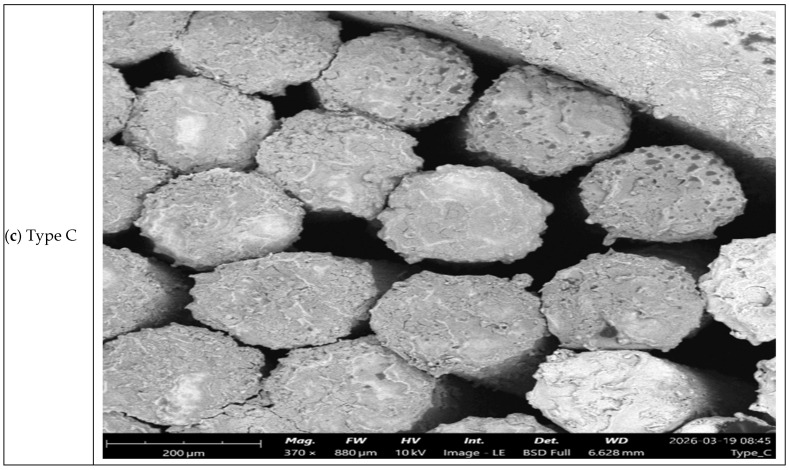
High-magnification SEM images of the crimp interface show conductor-to-conductor contact behavior for (**a**) Type A, (**b**) Type B, and (**c**) Type C configurations.

Further microstructural investigation of contact behavior was conducted using high-magnification SEM images, as shown in Figure 14. The results reveal geometry-dependent differences in conductor-to-conductor contact behavior, which are summarized in Table 10.

In the Type A configuration, there is continuous metallic contact between the strands, suggesting significant minimization of boundaries because of plastic deformation. In such a case, the strands become highly compacted. Conversely, in the Type B configuration, there is partial contact between the strands, which can be attributed to the existence of boundaries as well as the creation of voids between strands. This causes moderate compaction. In the Type C configuration, the strands have become clearly separated due to the development of voids.

This suggests poor deformation coupled with discontinuous contact interfaces. In this regard, the shift from continuous to discontinuous contact translates into a shift from a percolated conductive path network to a discontinuous network as depicted by an increase in electrical resistance [32]. According to Table 10 below, this shift correlates with high void fraction and low effective contact area that affect conductivity as well as load transfer capacity.

The SEM-derived deformation trends are also consistent with the FEM predictions regarding confinement behavior. The numerical analysis configurations that have been subjected to greater confinement tend to have a more compact conductor structure, together with greater continuity depicted in the SEM images. It is also important to note that these findings correlate with those of ANOVA, whereby crimp geometry appears to dominate temperature/humidity conditions.

The results collectively establish a strong and consistent relationship among crimp geometry, contact interface topology, and the functional performance of the crimped connection. Crimp geometry governs not only the mechanical and electrical performance of the joint but also the contact interface topology that underlies that performance.

### 3.3. Finite Element Modeling (FEM) Analysis

To shed light on the geometry-dependent degradation patterns found in both experiments and statistical studies, the crimped terminal deformation properties were studied using the finite element method (FEM). Consistent with the experimental approach, the conductor was excluded from the FEM model so that deformation could be attributed solely to crimp geometry. Therefore, the experimental analysis was carried out on crimped terminals without conductors, and the numerical simulation was carried out under the same conditions as well.

**Figure 15:**
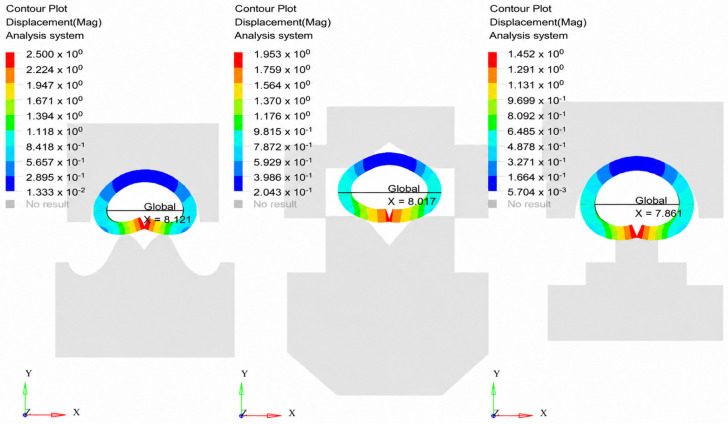
FEM displacement magnitude contours showing geometry-dependent deformation behavior of the crimped terminal under identical crimping load.

The objective of the numerical simulation was to measure deformation characteristics such as displacement distribution and deformation concentration around the crimped portion. Thus, the FEM output allows for the determination of qualitative deformation patterns based on crimp geometry. The FEM results reveal clear geometry-dependent differences in deformation response. The FEM findings coincide qualitatively with those obtained experimentally based on changes in electrical resistance, tensile strength, and microstructure [33]. Thus, it was concluded that deformation depends directly on crimp geometry. Figure 15 shows the displacement distribution from the numerical simulation.

The deformation distribution results obtained from the FEM simulation studies are illustrated in Figure 15. With an equally applied crimping force, the Type A geometry showed the highest deformation in the X-direction among all the tested geometries. Such a deformation mechanism also caused the strongest inward crimping on the terminal walls, hence leading to contraction of the inner cavity region. Without considering the inclusion of the conductor bundle in the simulation model, such a deformation trend suggests a higher confinement effect inside the crimp region. In contrast, both Type B and Type C geometries experienced smaller lateral expansion, hence forming larger residual cavity regions after crimping and producing less confinement effects compared to Type A.

As can be seen from the results presented in Table 11, there appeared to be a modest geometric correlation between the lateral expansion measurements performed experimentally and the X-direction displacement predictions made via FEM. Such correspondence does not indicate an extensive validation of the developed FEM model, nor does it confirm contact mechanics in the crimp assembly. Instead, it shows only that, using the simplified FEM model in this work, it was possible to capture the relative trends in deformations for different terminal geometries. Since the conductor bundle was not considered in the model, the FEM simulation results were taken here just as a qualitative illustration of relative deformation trends in this work.

Since the conductor bundle is not incorporated in the finite element model, neither the terminal–conductor interaction nor the inter-conductor contact mechanics are represented. In consequence, the effects of the interaction between the conductor and the terminal, as well as between the conductors themselves, are not considered in the modeling process. Hence, the findings obtained using finite element modeling cannot provide insight into any other behavior besides that of terminal deformation regarding the applied boundary conditions.

The simulated displacement fields and internal cavity variations therefore serve only as qualitative indicators of confinement behavior [34,35]. Though they help understand the geometric dependencies of deformation behavior, the underlying principles of electrical resistance behavior and mechanical loading can only be confirmed experimentally.

Thus, the role of the finite element model employed herein is to serve as an illustration of the behavior of the system based on qualitative findings.

### 3.4. Factorial Design and Statistical Analysis (ANOVA)

Factorial analysis of variance (ANOVA) was applied to assess the relative contributions of governing factors to degradation in the crimped bonding joints. This approach provides an analytical framework for quantifying the effects of multiple input parameters and their interactions within a single experimental design [36].

In the present study, factorial ANOVA was applied to evaluate the influence of crimp geometry and environmental exposure on relative electrical resistance change ΔR (%) and tensile strength variation ΔT (%). To increase the reliability of the results, three samples were tested under each experimental setting, and each sample had three readings taken for each condition. Although condition means are reported in tables and figures for clarity, ANOVA was conducted on raw replicate data rather than means, and all statistics were derived directly from these data sets.

The factorial ANOVA results presented in Table 12 indicate that the statistical model accounts for 99.96% of the observed variance, demonstrating excellent fit. This model is characterized by a small *p*-value (*p* < 0.001) and a high F-value (5885.58), which indicates that the chosen input variables significantly influence the relative resistance change, ΔR (%). The variance decomposition reveals that linear terms account for more than 99% of the total variance. Therefore, degradation behavior is influenced only slightly by other factors and parameter interactions. Crimp geometry emerges as the dominant factor, exerting a substantially greater influence on electrical degradation than exposure duration.

**Figure 16:**
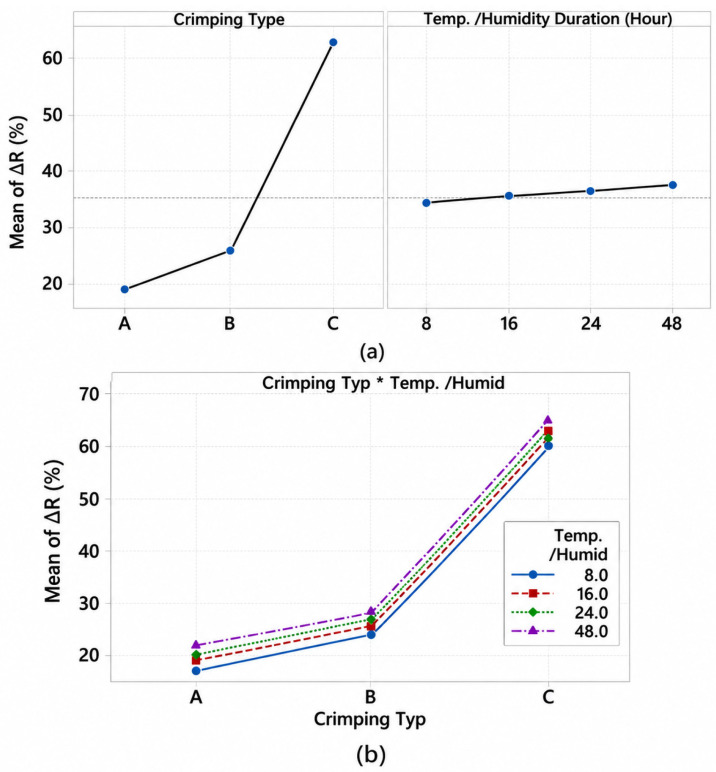
Statistical diagnostics for relative resistance change ΔR (%): (**a**) main effects plot illustrating the dominant influence of crimp geometry and exposure duration; (**b**) interaction plot showing the consistent degradation behavior across different crimping geometries under thermal–humidity cycles.

The primary effects graph shown in Figure 16a demonstrates the separate effect of each independent variable on relative resistance change. The sharpness of the slope for crimp geometry compared to the slight slope of the line representing exposure time corresponds with the findings obtained through the analysis of variance presented in the previous section and shown in Table 13. The significantly larger mean resistance growth in the case of Type C crimps suggests that this design is more sensitive to the external factors and might demonstrate lower interface stability.

Moreover, the interaction graph depicted in Figure 16b shows that the effect of exposure time is stable across different types of crimps. Since the lines representing the interactions do not show any intersections or divergences, it can be stated that while the actual magnitude of the damage depends on crimp geometry, the process of degradation itself is uniform in terms of time under the influence of thermal–humidity stress. These results underscore the critical role of initial crimp quality in establishing the long-term reliability of electrical contact interfaces.

The factorial ANOVA results for tensile strength variation (Table 14) indicate that the model accounts for 99.97% of the total variance. Crimp geometry alone accounts for 93.30% of the total variation in tensile strength—the largest single contribution observed in this study. In contrast, the environmental exposure time, although statistically significant, had a relatively lower impact when compared to crimping geometry on the mechanical breakdown of the joint. These results confirm that the initial density and deformation patterns associated with the crimp geometries, where Type A performs better than Type C due to its oval form, were critical factors to mechanical degradation [37].

The high F-value (6392.94) confirms the statistical significance of the factors considered; however, it does not imply predictive accuracy beyond the conditions tested. Taken together, the results demonstrate that crimp geometry is the most influential factor governing joint mechanical properties.

**Figure 17:**
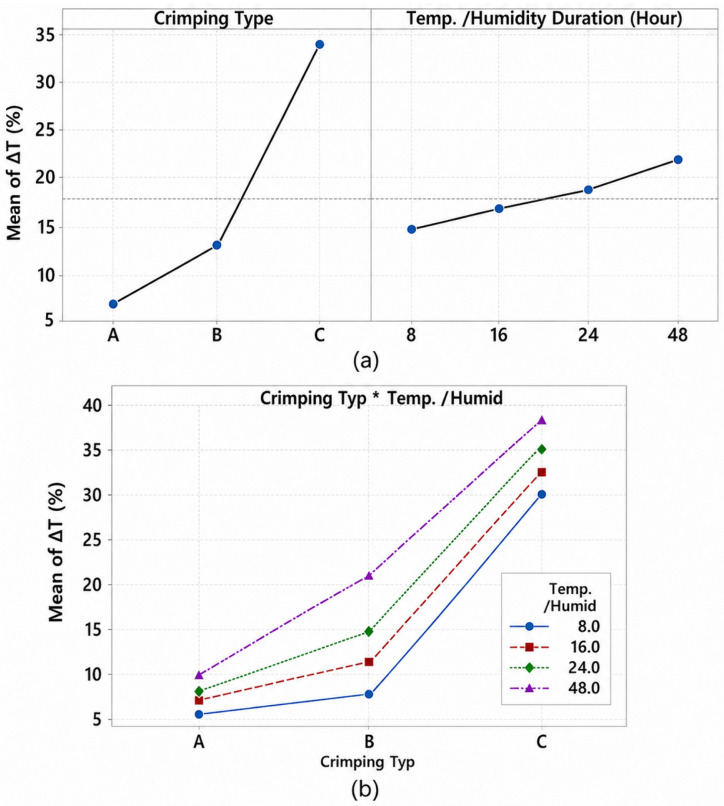
Statistical diagnostics for tensile strength variation ΔT (%): (**a**) main effects plot demonstrating the governing influence of crimp geometry; (**b**) interaction plot showing the consistent mechanical behavior across different configurations under environmental stress.

The main effects plot in Figure 17a confirms the dominant role of crimp geometry in governing joint mechanical stability. In particular, the large slope that corresponds to the crimping factor demonstrates its importance in the degradation process compared to the smaller slope, which is due to the time of exposure to the environment. For instance, the high level of degradation in tensile strength in Type C crimps implies poor stress distribution and fatigue in the materials, unlike in the case of single indentation Type A crimps.

Additionally, the interaction plot depicted in Figure 17b shows that the interaction effect between the crimping type and exposure period is statistically insignificant because the two plots are parallel. This confirms that a mechanical degradation threshold is established primarily by the crimping process itself, independent of exposure duration.

The three-factor factorial analysis examining the combined effects of chemical exposure type, crimp geometry, and short-circuit current is summarized in Table 15. All figures shown represent the average of three independent tests.

To validate the statistical significance, three different experimental setups were performed to establish consistency of the findings. The data displayed in Table 15 are the averages of the three datasets. These factors were selected to characterize the influence of fuel, hydraulic fluid, and de-icing fluid on the crimped bonding joint.

The generalized factorial ANOVA results, which assessed the relative change in resistance percentage ΔR (%) while considering the interaction of chemical exposure, crimp shape, and short-circuit current, are presented in Table 16 below. It can be noted that the model is well-fitted since the R^2^ coefficient equals 0.9993 and the F-statistics reaches 3185.49. The model fit is excellent (R^2^ = 0.9993; F = 3185.49; *p* < 0.001), confirming that the selected independent variables adequately explain the observed electrical degradation. In terms of the variance breakdown, one may observe that the most important factor is “Chemical exposure Type” with the explained share of 55.3%.

In a factorial ANOVA setting, a two-way interaction refers to the situation where the influence of one variable on the response depends on the level of another variable. For example, the Chemical Exposure Type by crimping geometry interaction (3.09%, Table 16) means that the additional ΔR (%) caused by any fluid is not the same for all crimp designs because Skydrol has an elevated effect on the resistance increase for Type C as opposed to Type A, due to the reduced contact density allowing more fluid intrusion into the gap. Another example of a two-way interaction would be the Chemical Exposure Type by short-circuit current (3.05%), meaning that each type of fluid causes an increased electrical effect to different degrees depending on the level of applied current, reflecting thermal-assisted surface film development at higher current densities. A three-way interaction, on the other hand, means that a two-way interaction effect depends on the level of the third factor. In this case, Chemical Exposure Type by crimping geometry by short-circuit current (4.01%) represents an interaction where fluid aggressiveness, contact density, and resistive heating have an interactive effect on the electrical degradation response.

**Figure 18:**
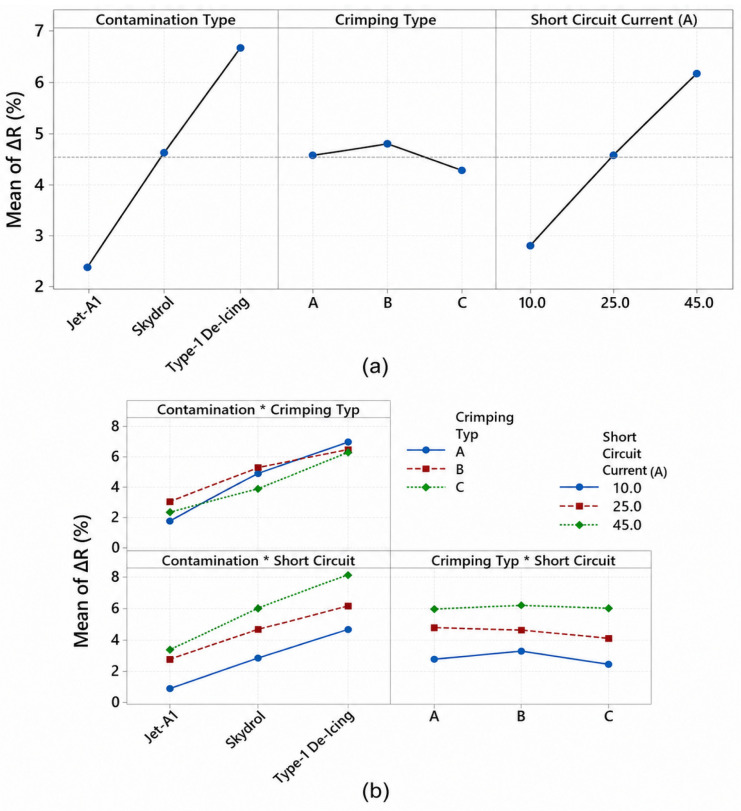
Statistical diagnostics for relative resistance change ΔR (%) under combined stressors: (**a**) main effects plot identifying the primary influence of chemical exposure type; (**b**) interaction plot revealing the synergistic behavior between chemical exposure, crimp geometry, and electrical load.

The individual influence of contaminant type, crimp geometry, and short-circuit current on the relative resistance variation ΔR (%) is shown in the plot for the main effects shown in Figure 18a. As seen from the percentage contribution of 55.3%, there is a great effect of contaminant types and resistance changes under some chemicals, which proves that the dominating factor in the electrical degradation process is the contaminant type.

Based on these results, the dominant degradation mechanism shifts depending on the loading condition. While in the case of mechanical/electromechanical loading, the crimp geometry is the factor defining the contact density and electrical contact deformation, in the case of chemical/electrical testing, the surface mechanisms are observed; that is why crimp geometry becomes less important. Nevertheless, observable trends associated with both geometry and short-circuit current persist. An increase in the electric current leads to heating due to resistive heating. Therefore, both contaminants and currents affect the importance of geometry in terms of electrical resistance, but indirectly, since they affect the first contact point.

The interaction plot in Figure 18b illustrates the three-factor interactions. The interaction plot shows the different lines, indicating that although there is no accumulative effect in terms of contaminant types, there can be an enhancement of the effects caused by the crimp geometry or current used for the contact interfaces. As per this study, each point represents the average of three experiments, hence the validity of the interaction between these three factors.

Factorial ANOVA is used to assess the influence of chemical exposure type, crimping geometry, and short-circuit current on the ΔT (%) variation in tensile strength in Table 17 below. The model achieves a total explained variance of 99.12% (F = 232.91; *p* < 0.001), confirming excellent fit. Crimp geometry is the most influential factor, accounting for 61.87% of the variance in ΔT (%). On the contrary, chemical exposure type and short-circuit current exert minimal influences on the tensile strength. Factors with near-zero contribution rates in Table 17 confirm that plastic deformation—governed by crimp geometry—is the primary driver of tensile strength variation, with chemical and electrical factors playing secondary roles. Additionally, to improve the accuracy of the experiment, average data was used for analysis purposes, which was taken from three separate test sets.

The interaction effects in Table 17 are of particular mechanistic significance. The synergistic effect in this complex mechanism can be defined from the point of view of the fact that the interaction of the crimp geometry and the contamination type determines the vulnerability of the connections to chemical degradation, which, in turn, depends on the mechanical connection quality. In more detail, it means that the tensile strength associated with the high quality of crimping geometries (Type A) does not depend on the chemical, while the low tensile strength associated with the poor quality of geometries (Type C) is decreased faster under the influence of aggressive chemicals. The different trends show that crimp geometry acts as a physical barrier, limiting the liquid access in the high-quality geometries and enhancing it in the low-quality geometries, which results in stress concentration and corrosion initiation. As seen from the Table 17, the effect of interaction is larger than each of the two independent variables. This means that interaction is a key factor affecting mechanical degradation. Further aging trials are warranted to enable failure prediction under extended exposure conditions.

Within factorial ANOVA, a two-way interaction is a situation in which the effect of one variable on the outcome depends on the level of a second variable. The two-way interaction between chemical exposure type and crimping type (36.18% for ΔT; Table 17) implies that tensile degradation caused by the fluid depends on the crimp geometry, such that Skydrol causes greater tensile loss in Type B geometry compared to Type A geometry due to differential strand engagement. A three-way interaction suggests that the two-way interaction is different depending on the level of the third variable. In the present data set, the three-way interaction between chemical exposure type, crimping type, and short-circuit current explains 0.00% of ΔT variation, confirming that current does not modify the relationship between fluid and geometry for mechanical degradation. In Table 16, DF stands for the degrees of freedom, Seq SS refers to the sequential sum of squares, Adj SS means the adjusted sum of squares, and Adj MS is the adjusted mean squares.

**Figure 19:**
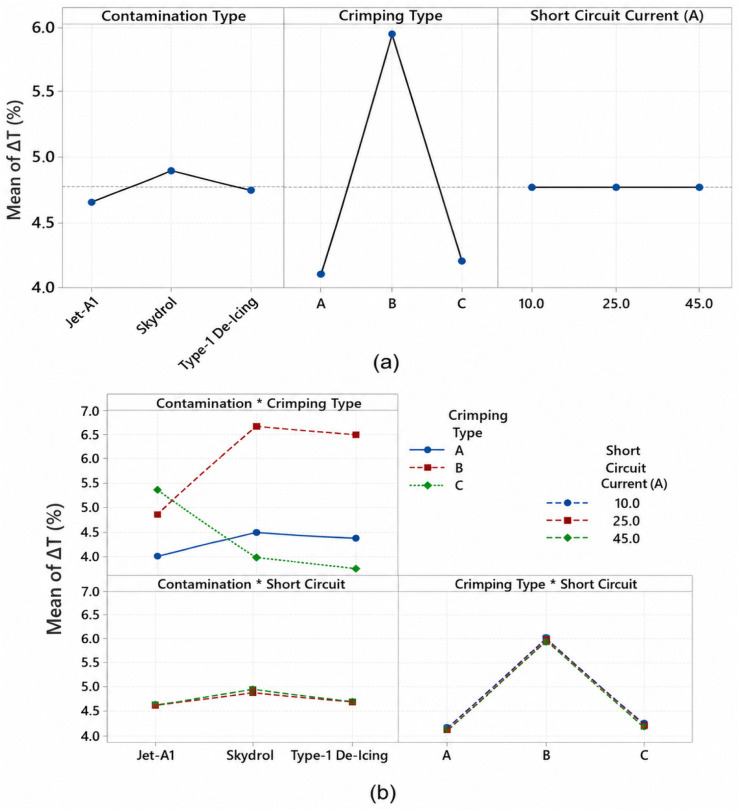
Statistical diagnostics for tensile strength variation ΔT (%) under multi-stress conditions: (**a**) main effects plot confirming the dominance of crimp geometry; (**b**) interaction plot revealing the complex synergistic relationships between mechanical design and environmental/electrical stressors.

The main effects plot for the deviation in tensile strength ΔT (%) is shown in Figure 19a, which acts as an additional confirmation of the ANOVA findings presented in Table 17. The steep gradient of the crimping geometry factor, compared with the relatively stable curves of the other two factors, namely chemical exposure and short-circuit current, verifies the primary role of the initial mechanical design in establishing the ability of the connection to withstand stress. Although the Type A design, having only one indentation, retains the highest value of residual strength, the significant reduction in the stress-resisting ability of the connection, as demonstrated by the Type B design (hexagonal form), is evident.

Figure 19b shows the interaction graph that confirms the strong two-way interactions discovered in the factorial ANOVA test. The non-parallel curves for crimp geometry and chemical exposure type demonstrate that chemical degradation of mechanical performance is not uniform across geometries; it is strongly modulated by crimp compaction quality. Also, a densely packed crimp geometry acts as a hindrance to the contaminants entering the material, thus having a detrimental effect on its load-bearing capabilities. On the other hand, a less densely packed crimp geometry aids the capillary action of contaminants and therefore has an increased detrimental effect. Every data point on the graph represents the average value from three different replicate tests, meaning that the geometry × chemical exposure interaction is statistically significant under controlled conditions.

The factorial design and ANOVA used in this study reveal the factors that dictate the electrical and mechanical performance capabilities of the aviation connectors with an accuracy of greater than 99% (R^2^ > 0.99). This suggests that there is a statistical relationship between the selected factors and the measured responses, as opposed to the general predictive model. While the past literature has mostly focused on studying the environmental effects and degradation drivers for aviation connectors using isolated stressors, this analysis focuses on studying an uncharted area, which is the crimp geometry as the main “degradation driver.” The statistical analysis conducted shows that the main factor within the scope of analysis is indeed the crimp geometry as a “degradation driver.” The mechanical variance contribution of 61.87% confirms that long-term connection reliability is predominantly determined by the deformation state established during manufacturing, rather than by external stressors. These findings further support the interpretation that crimp quality functions not only as a mechanical interface but also as a physical barrier against environmental ingress. High bidirectional interaction rates found through the various statistical analyses show how complicated the mechanism of degradation is under extreme environmental conditions of aviation engine nacelles. High-density geometries such as Type A demonstrate significant resistance to environmental factors, whereas low-density geometries such as Type C are susceptible to fluid ingress and capillary-driven degradation—effects that combine with thermal and electrical stress to produce a multiplicative degradation response.

## 4. Conclusions

In this study, the geometry-driven degradation behavior of crimped bonding assemblies under coupled chemical–environmental loading was investigated. The four research objectives stated in Section 1 are addressed below.

(i) Decoupling of crimp geometry from all other assembly variables: Under identical material, terminal, and processing conditions, the three crimp geometries (Types A, B, and C) produced distinctly different electrical and mechanical responses. Specifically, when subjected to environmental exposures, Geometry A (W form) showed the least increase in electrical resistance and maintained the tensile strength, while Geometry C (oval form) showed the worst degradation among all geometries. As indicated by the failure mode classification in Table 7, the geometries also differ significantly in failure mechanism. The failure modes recorded are as follows: CWF in Type A, MMF in Type B, and CPO in Type C. The relatively low tensile strength of Type C is therefore attributable primarily to the insufficient mechanical interlock of the conductor strands within the oval crimp barrel.
(ii) Quantification of crimp geometry contributions to degradation during thermal–humidity exposure: In the replicated full-factorial ANOVA, crimp geometry accounted for 99.56% of the variance in ΔR (%) and 93.30% of the variance in ΔT (%). In comparison, the effect of exposure time was smaller than 0.39% and 5.56%, respectively (*p* < 0.001 for both). Consequently, regarding the examined conditions of thermal–humidity exposure, the long-term behavior of the bonding joint depends mainly on crimp geometry, that is, the mechanical assembly as originally assembled.
(iii) Shift in degradation dominance under combined chemical and electrical loading: In the presence of a combination of a chemical stressor and an electrical current, chemical stress becomes the main determinant of the degradation processes (55.35%), followed by the influence of electric current (33.24%), whereas the significance of crimp geometry decreases to (0.73%). Skydrol hydraulic fluid produced the greatest electrical degradation, which is consistent with its high polarity and surface-active properties [24,25]; however, identification of the specific reaction products formed at the interface is beyond the scope of this study and warrants dedicated future investigation. At the same time, crimp geometry continues to be the dominating factor for mechanical degradation processes (61.87%). These results indicate that each degradation process is characterized by its own factors influencing the process, i.e., crimp geometry acts as a barrier to chemically mediated electrical degradation while remaining the principal determinant of mechanical load transfer through plastic deformation. Interactions, such as chemical stress × crimp geometry (36.18% for ΔT%), can be treated as multiplicative in nature.
(iv) Interpretation of the results from the perspective of micromechanics and numerics and the model’s ability to predict the behavior of different geometries under identical conditions: Scanning Electron Microscopy (SEM) demonstrated a significant effect of the crimp geometry on the number of voids (0.46% for Type A, 7.98% for Type B, and 14.25% for Type C) and on the contact type (fragmented contact vs. continuous contact). Finite element modeling qualitatively reproduced the geometry-dependent tendencies regarding deformation of samples, especially the differences between the types of crimps in terms of deformation and confinement (Type A > Type B > Type C). Since the interaction of the conductor bundle and conductor-to-terminal contact has not been modeled, the FEM calculations were used for qualitative explanation of trends rather than as a tool for prediction or assessment of the electrical and mechanical properties. From the combination of statistics, micromechanical, and numerical results, one can conclude about the significant effect of the crimp geometry on contact type, void creation, and deformation/confinement, which are strongly correlated with mechanical and electrical characteristics. Therefore, the current research is a starting point for a framework for studying degradation behavior based on geometry.

These four goals together show that crimp geometry is not just a variable in assembly but acts as a degradation control mechanism whose relevance depends upon the stress type involved. Crimp geometry is the dominant factor under thermal–humidity aging and retains a significant role even under multi-stress conditions. This study also highlights that crimp geometry acts as a physical shield against any chemical attack, and its efficiency depends on mechanical compression at the bond site. Future research will involve modeling for extended periods of aging, testing with other chemical substances, and finite element models incorporating conductors based on contact mechanics, leading to the development of a predictive lifetime model.

## Figures and Tables

**Table 1:** Controlled material and assembly parameters used in experimental specimens.

Parameter	Specifications	Experimental Status
Cable Type	ASNE0092	Fixed
Conductor Structure	Multi-stranded copper (core + braid)	Fixed
Conductor Cross-Section	13.0 mm^2^	Fixed
Plating Type	Nickel-plated	Fixed
Terminal Type	NSA936020-05N (Nickel-plated)	Fixed
Terminal Material	Copper	Fixed
Terminal Plating	Nickel	Fixed
Terminal Thickness	1.52 mm	Fixed
Recommended Lengths (mm)	200 (±2 tolerance)	Fixed
Stabilization Condition	23 ± 2 °C, 50 ± 10% RH (24 h)	Fixed pre-test condition
Operating Temperature (Nickel-plated)	Maximum 250 °C	Fixed
Crimping Force	Nominal crimping force: 200 N (verified using calibrated load-cell measurement)	Fixed
Variable Parameter	Crimp geometry (Types A, B, and C)	Investigated variable

**Table 2:** Selected geometric parameters measured from cross-sectional profiles of the investigated crimp geometries.

Parameter	Type A (mm)	Type B (mm)	Type C (mm)
Crimp Height	5.10 ± 0.04	5.45 ± 0.04	6.40 ± 0.04
Crimp Width	8.209 ± 0.04	8.099 ± 0.04	7.931 ± 0.04
Splice/Base Thickness	0.0253 ± 0.003	0.13208 ± 0.003	0.2756 ± 0.003

**Table 3:** Main technical specifications of the Phenom XL G3 SEM [23].

Parameter	Specification
Instrument Model	Thermo Scientific Phenom XL
Electron Source	CeB_6_ (Cerium hexaboride)
Accelerating Voltage	4.8–20.5 kV
Maximum Sample Size	100 mm × 100 mm
Resolution	9–14 nm
Detectors	Backscattered electron (BSE), optional SE/EDS
Imaging Time	<60 s (typical)
Sample Stage	Motorized, multi-sample capability

**Table 4:** Material properties of upper and lower jaws at room temperature.

Property	Symbol	Value	Unit
Young’s Modulus	E	210,000	MPa
Poisson Ratio	ν	0.30	-

**Table 5:** Mechanical properties of annealed copper (C11000).

Property	Symbol	Value	Unit
Young’s Modulus	E	110,000	MPa
Poisson Ratio	ν	0.34	-
Yield Strength (0.2% offset)	σ_y_	69–75	MPa
Ultimate Tensile Strength	σ_u_	200–220	MPa
Elongation at Break	ε	40–50	%

**Table 6:** The true stress–true strain data used in the numerical model [24].

True Stress [MPa]	True Strain
70	0.000
100	0.020
130	0.050
160	0.100
185	0.200
205	0.350
215	0.450

**Table 7:** Failure-mode classification of crimped bonding specimens for each crimp geometry and exposure condition (CWF—conductor wire fracture; MMF—mixed-mode failure; and CPO—conductor pull-out/slippage).

Crimp Geometry	Baseline (As-Crimped)	Thermal–Humidity (48 h)	Jet A-1 + 45 A	Skydrol + 45 A	Type I De-icing + 45 A
Type A (W form)	CWF	CWF	CWF	CWF	CWF
Type B (symmetric)	MMF	MMF	MMF	MMF	MMF
Type C (oval form)	CPO	CPO	CPO	CPO	CPO

**Table 8:** Comparison of tensile strength degradation for different chemical exposure types and crimp geometries.

Chemical Exposure Type	Crimping Geometry	T_0_ (N)	T_1_ (N)	ΔT (%)
Jet A-1	A	1118	1075	3.85
Jet A-1	B	641.8	611	4.8
Jet A-1	C	73.8	69.9	5.28
Skydrol	A	1118	1070	4.29
Skydrol	B	641.8	599	6.67
Skydrol	C	73.8	71	3.79
Type I De-Icing	A	1118	1071	4.20
Type I De-Icing	B	641.8	600	6.51
Type I De-Icing	C	73.8	71.2	3.52

**Table 9:** Quantitative evaluation of the void fraction and the corresponding contact area in the crimp region for different crimp geometries, based on SEM image analysis.

Crimp Geometry	Void (%)	Contact (%)
Type A	0.46	99.54
Type B	7.98	92.02
Type C	14.25	85.75

**Table 10:** Qualitative comparison of contact behavior based on SEM observations.

Crimp Geometry	Plastic Deformation	Contact Continuity	Void Distribution
Type A	High	Continuous	Minimal
Type B	Moderate	Partial	Distributed
Type C	Low	Discontinuous	Significant

**Table 11:** Comparison of experimentally measured lateral expansion and FEM-predicted displacement in the X-direction for different crimp geometries under conductor-free conditions.

Crimp Geometry	Experimental Lateral Expansion (mm)	FEM Displacement (mm)
Type A	8.209	8.121
Type B	8.099	8.017
Type C	7.931	7.861

**Table 12:** Reference electrical resistance and tensile strength values measured before and after temperature/humidity exposure, together with the derived relative resistance change ΔR (%) and tensile strength variation ΔT (%) for each crimping geometry and exposure duration (values represent the mean of three independent replicates (*n* = 3); SD: standard deviation).

Experiment No.	Crimping Geometry	Temperature/Humidity Duration (Hour)	R_0_ (mΩ)	R_1_ (mΩ)	T_0_ (N)	T_1_ (N)	ΔR (%)	ΔT (%)	SD ΔT
1	A	8	0.664	0.779	1118	1053	17.4	5.8	±0.053
2	A	16	0.664	0.789	1118	1034	18.9	7.5	±0.044
3	A	24	0.663	0.792	1117	1030	19.4	7.8	±0.061
4	A	48	0.663	0.802	1114	1009	20.9	9.4	±0.087
5	B	8	0.885	1.106	641	588	24.9	8.3	±0.217
6	B	16	0.885	1.111	642	569	25.6	11.4	±0.207
7	B	24	0.884	1.121	642	552	26.8	14.0	±0.315
8	B	48	0.885	1.132	644	518	27.9	19.6	±0.236
9	C	8	0.943	1.518	79.8	55.8	60.9	30.1	±0.240
10	C	16	0.944	1.528	79.7	54	61.9	32.2	±0.098
11	C	24	0.944	1.536	80	52	62.7	35.0	±0.789
12	C	48	0.944	1.551	80	50	64.3	37.5	±0.962

**Table 13:** General factorial ANOVA analysis and results for relative resistance change ΔR (%) as a function of crimp geometry and thermal–humidity exposure duration.

Source	DF	Seq SS	Contribution	Adj SS	Adj MS	F-Value	*p*-Value
Model	11	12,984.2	99.96%	12,984.2	1180.39	5885.58	<0.001
Linear	5	12,983.3	99.96%	12,983.3	2596.66	12,947.32	<0.001
Crimping Geometry	2	12,932.3	99.56%	12,932.3	6466.14	32,241.13	<0.001
Temp./Humidity Duration (Hour)	3	51	0.39%	51	17	84.78	<0.001
2-Way Interactions	6	1	0.01%	1	0.16	0.8	0.581
CrimpingType*Temp./Humidity Duration (Hour)	6	1	0.01%	1	0.16	0.8	0.581
Error	24	4.8	0.04%	4.8	0.2		
Total	35	12,989.1	100.00%				

Note: In Table 13, DF denotes the degrees of freedom, Seq SS represents the sequential sum of squares, Adj SS indicates the adjusted sum of squares, and Adj MS corresponds to the adjusted mean squares.

**Table 14:** General factorial ANOVA analysis and the results for tensile strength variation ΔT (%) illustrating the dominance of crimp geometry over environmental exposure duration.

Source	DF	Seq SS	Contribution	Adj SS	Adj MS	F-Value	*p*-Value
Model	11	4844.43	99.97%	4844.43	440.4	6392.94	<0.001
Linear	5	4790.84	98.86%	4790.84	958.17	13,908.88	<0.001
Crimping Geometry	2	4521.56	93.30%	4521.56	2260.78	32,817.78	<0.001
Temp./Humidity Duration (Hour)	3	269.28	5.56%	269.28	89.76	1302.95	<0.001
2-Way Interactions	6	53.59	1.11%	53.59	8.93	129.66	<0.001
Crimping Geometry*Temp./Humidity Duration (Hour)	6	53.59	1.11%	53.59	8.93	129.66	<0.001
Error	24	1.65	0.03%	1.65	0.07		
Total	35	4846.08	100.00%				

Note: In Table 14, DF denotes the degrees of freedom, Seq SS represents the sequential sum of squares, Adj SS indicates the adjusted sum of squares, and Adj MS corresponds to the adjusted mean squares.

**Table 15:** Experimental factorial design framework for different chemical exposure types and average values derived from three independent experimental replicates.

Chemical Exposure Type	Crimping Geometry	Applied Short-Circuit Current (A)	R_0_ (mΩ)	R_1_ (mΩ)	T_0_ (N)	T_1_ (N)	ΔR (%)	ΔT (%)	SD ΔT
Jet-A1	A	10	1.4	1.41	1118	1075	0.71	3.85	±1.43
Jet-A1	A	25	1.43	1.46			2.10		
Jet-A1	A	45	1.5	1.53			2.00		
Jet-A1	B	10	1.6	1.61	641.8	611	0.63	4.8	±1.37
Jet-A1	B	25	1.6	1.65			3.12		
Jet-A1	B	45	1.6	1.67			4.37		
Jet-A1	C	10	2.33	2.36	73.8	69.9	1.29	5.28	±1.35
Jet-A1	C	25	2.33	2.4			3.00		
Jet-A1	C	45	2.35	2.43			3.40		
Skydrol	A	10	1.33	1.36	1118	1070	2.26	4.29	±1.11
Skydrol	A	25	1.33	1.4			5.26		
Skydrol	A	45	1.43	1.53			6.99		
Skydrol	B	10	1.53	1.6	641.8	599	4.58	6.67	±1.68
Skydrol	B	25	1.56	1.63			4.49		
Skydrol	B	45	1.66	1.76			6.02		
Skydrol	C	10	2.36	2.4	73.8	71	1.69	3.79	±1.60
Skydrol	C	25	2.4	2.5			4.17		
Skydrol	C	45	2.4	2.53			5.42		
Type I De-Icing	A	10	1.36	1.43	1118	1071	5.15	4.20	±1.75
Type I De-Icing	A	25	1.46	1.56			6.85		
Type I De-Icing	A	45	1.56	1.7			8.97		
Type I De-Icing	B	10	1.53	1.6	641.8	600	4.58	6.51	±2.10
Type I De-Icing	B	25	1.6	1.7			6.25		
Type I De-Icing	B	45	1.66	1.8			8.43		
Type I De-Icing	C	10	2.36	2.46	73.8	71.2	4.24	3.52	±2.02
Type I De-Icing	C	25	2.43	2.56			5.35		
Type I De-Icing	C	45	2.43	2.66			9.47		

Note: T_0_ values represent the average tensile strength of three samples measured prior to chemical exposure and current exposure. Since these baseline values are identical for each crimping geometry, they are reported once and omitted for repeated current levels. T_1_ values represent the average tensile strength of the same samples after chemical exposure and current exposure.

**Table 16:** General factorial ANOVA and the results for relative resistance change ΔR (%) as a function of chemical exposure, crimp configuration, and short-circuit current loading.

Source	DF	Seq SS	Contribution	Adj SS	Adj MS	F-Value	*p*-Value
Model	26	457.412	99.93%	457.412	17.593	3185.49	<0.001
Linear	6	408.825	89.32%	408.825	68.138	12,337.53	<0.001
Chemical Exposure Type	2	253.334	55.35%	253.334	126.667	22,935.34	<0.001
Crimping Geometry	2	3.358	0.73%	3.358	1.679	303.97	<0.001
Short-Circuit Current (A)	2	152.134	33.24%	152.134	76.067	13,773.27	<0.001
2-Way Interactions	12	30.237	6.61%	30.237	2.52	456.25	<0.001
Chemical Exposure Type*Crimping Geometry	4	14.144	3.09%	14.144	3.536	640.25	<0.001
Chemical Exposure Type*Short-Circuit Current (A)	4	13.981	3.05%	13.981	3.495	632.9	<0.001
Crimping Geometry*Short-Circuit Current (A)	4	2.112	0.46%	2.112	0.528	95.6	<0.001
3-Way Interactions	8	18.349	4.01%	18.349	2.294	415.3	<0.001
Chemical Exposure Type*Crimping Geometry*Short-Circuit Current (A)	8	18.349	4.01%	18.349	2.294	415.3	<0.001
Error	54	0.298	0.07%	0.298	0.006		
Total	80	457.71	100.00%				

Note: In Table 16, DF denotes the degrees of freedom, Seq SS represents the sequential sum of squares, Adj SS indicates the adjusted sum of squares, and Adj MS corresponds to the adjusted mean squares.

**Table 17:** General factorial ANOVA and the results for tensile strength variation ΔT (%) showcasing the predominant influence of crimp geometry under combined chemical and electrical stress conditions.

Source	DF	Seq SS	Contribution	Adj SS	Adj MS	F-Value	*p*-Value
Model	26	97.376	99.12%	97.376	3.7452	232.91	<0.001
Linear	6	61.8324	62.94%	61.8324	10.3054	640.87	<0.001
Chemical Exposure Type	2	1.0446	1.06%	1.0446	0.5223	32.48	<0.001
Crimping Geometry	2	60.7878	61.87%	60.7878	30.3939	1890.13	<0.001
Short-Circuit Current (A)	2	0	0.00%	0	0	0	1
2-Way Interactions	12	35.5436	36.18%	35.5436	2.962	184.2	<0.001
Chemical Exposure Type*Crimping Geometry	4	35.5436	36.18%	35.5436	8.8859	552.59	<0.001
Chemical Exposure Type*Short-Circuit Current (A)	4	0	0.00%	0	0	0	1
Crimping Geometry*Short-Circuit Current (A)	4	0	0.00%	0	0	0	1
3-Way Interactions	8	0	0.00%	0	0	0	1
Chemical Exposure Type*Crimping Geometry*Short-Circuit Current (A)	8	0	0.00%	0	0	0	1
Error	54	0.8683	0.88%	0.8683	0.0161		
Total	80	98.2444	100.00%				

Note: In Table 17, DF denotes the degrees of freedom, Seq SS represents the sequential sum of squares, Adj SS indicates the adjusted sum of squares, and Adj MS corresponds to the adjusted mean squares.

## Data Availability

The original contributions presented in this study are included in the article. Further inquiries can be directed to the corresponding author.

## Abbreviations

ANOVA	Analysis of Variance
AS	Aerospace Standard
ASNE	Aerospace Standard Nickel-Plated
Cu	Copper
Cu–Be	Copper–Beryllium Alloy
DO-160G	Environmental Conditions and Test Procedures for Airborne Equipment
EASA	European Union Aviation Safety Agency
EDS	Energy Dispersive Spectroscopy
FAA	Federal Aviation Administration
FEM	Finite Element Method
ISO	International Organization for Standardization
MIL-DTL	Military Detail Specification
MIL-STD	Military Standard
RH	Relative Humidity
RTCA	Radio Technical Commission for Aeronautics
SAE	Society of Automotive Engineers
SEM	Scanning Electron Microscopy
ΔR (%)	Percentage Change in Electrical Resistance
ΔT (%)	Percentage Change in Tensile Strength
N	Newton
R_0_	Electrical Resistance Before Exposure
R_1_	Electrical Resistance After Exposure
T_0_	Tensile Strength Before Exposure
T_1_	Tensile Strength After Exposure
